# Mechanism of Nitrone Formation by a Flavin-Dependent Monooxygenase

**DOI:** 10.1021/acs.biochem.3c00656

**Published:** 2024-05-23

**Authors:** Sydney B. Johnson, Hao Li, Hannah Valentino, Pablo Sobrado

**Affiliations:** † Department of Biochemistry, Virginia Tech, Blacksburg, Virginia 24061, United States; ‡ Center of Drug Discovery, Virginia Tech, Blacksburg, Virginia 24061, United States

## Abstract

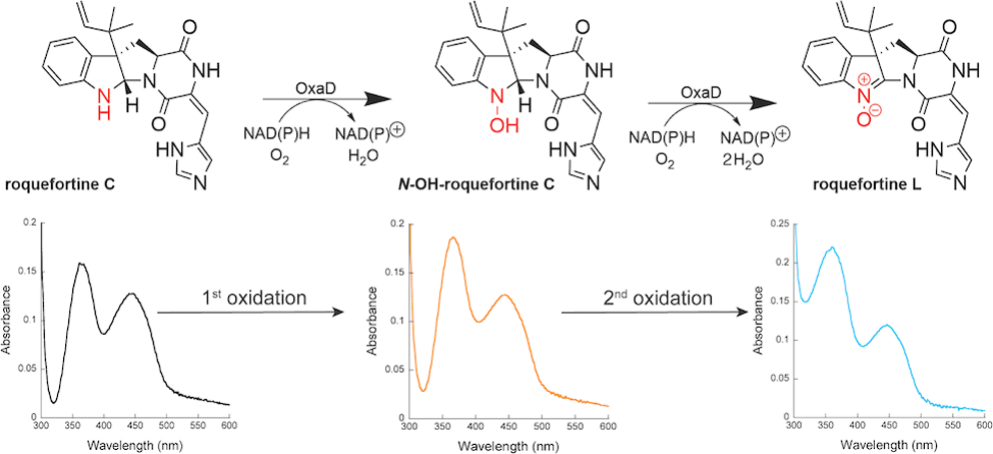

OxaD is a flavin-dependent monooxygenase (FMO) responsible for catalyzing the oxidation of an indole nitrogen atom, resulting in the formation of a nitrone. Nitrones serve as versatile intermediates in complex syntheses, including challenging reactions like cycloadditions. Traditional organic synthesis methods often yield limited results and involve environmentally harmful chemicals. Therefore, the enzymatic synthesis of nitrone-containing compounds holds promise for more sustainable industrial processes. In this study, we explored the catalytic mechanism of OxaD using a combination of steady-state and rapid-reaction kinetics, site-directed mutagenesis, spectroscopy, and structural modeling. Our investigations showed that OxaD catalyzes two oxidations of the indole nitrogen of roquefortine C, ultimately yielding roquefortine L. The reductive-half reaction analysis indicated that OxaD rapidly undergoes reduction and follows a “cautious” flavin reduction mechanism by requiring substrate binding before reduction can take place. This characteristic places OxaD in class A of the FMO family, a classification supported by a structural model featuring a single Rossmann nucleotide binding domain and a glutathione reductase fold. Furthermore, our spectroscopic analysis unveiled both enzyme–substrate and enzyme–intermediate complexes. Our analysis of the oxidative-half reaction suggests that the flavin dehydration step is the slow step in the catalytic cycle. Finally, through mutagenesis of the conserved D63 residue, we demonstrated its role in flavin motion and product oxygenation. Based on our findings, we propose a catalytic mechanism for OxaD and provide insights into the active site architecture within class A FMOs.

Flavoenzymes are versatile catalysts capable of participating in a wide range of reactions, with the flavin prosthetic group assuming roles as an electrophile and nucleophile and forming covalent adducts with substrates.^1−3^ Among flavoenzymes are the flavin-dependent monooxygenases (FMOs) which represent a large enzyme family that is dedicated to incorporating a single oxygen atom from molecular oxygen into a variety of substrates.^3,4^ The FMO family is classified based on structural characteristics, substrate preferences, and modes of flavin reduction, leading to eight subclasses (A–H).^4,5^ Class A FMOs, characterized by the requirement for substrate binding prior to reduction, exhibit two distinctive structural features: (1) a Rossmann nucleotide-binding domain and (2) a glutathione reductase fold.^4^ Class A members typically catalyze the single oxidation reactions of an aromatic carbon and can directly utilize NAD(P)H to reduce the flavin, eliminating the need for an additional enzyme reductase.^4^ OxaD, a recently identified class A FMO, stands out among other members of its class by catalyzing multiple N-oxidations of an indolic nitrogen found in a prenylated alkaloid from the marine-derived fungus *Penicillium oxalicum* F30.^6^ Previous research on OxaD suggested that it catalyzes two successive oxidations of roquefortine C, ultimately yielding roquefortine L, which includes a nitrone functional group, where OxaD is an active participant in its formation (Scheme 1).^6^ The biosynthesis of nitrones positions OxaD as a promising candidate for green chemistry applications due to the valuable electrophilic properties that nitrones offer, particularly in challenging organic synthesis reactions such as cycloadditions.^7,8^

**Scheme 1:**
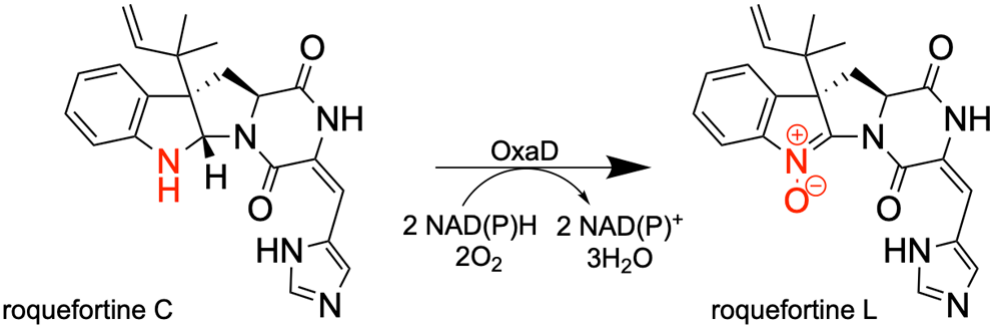
OxaD Reaction Scheme OxaD catalyzes the 4-electron oxidation of roquefortine C to yield roquefortine L.

Roquefortine C was initially isolated and identified from *Penicillium roqueforti*.^9^ This compound has demonstrated the ability to deactivate cytochrome P450s in the liver and kidneys, likely through binding to the heme in the active site.^10^ Other studies have reported cytotoxic, immunotoxic, neurotoxic, and lung inflammatory effects in mice exposed to roquefortine C.^11−14^ The product of the OxaD reaction, roquefortine L, is converted into glandicoline B by a cytochrome P450 monooxygenase (OxaH), which is further processed into melegranin, and oxaline by two *S*-adenosyl methionine (SAM)-dependent methyltransferases (OxaG and OxaC, respectively).^6,15−17^ Melegranin and oxaline have exhibited anticancer properties against human breast and leukemia cancer cell lines, with oxaline and glandicoline B also displaying antibacterial effects.^18−20^

Previous investigations into OxaD have primarily focused on its substrate scope, providing limited knowledge regarding its catalytic mechanism.^6^ Our objective was to uncover the catalytic mechanism of this unique FMO, offering a foundation for both biomedical and chemical synthetic applications. To achieve this goal, we employed a combination of rapid-reaction and steady-state kinetics, structural modeling, and site-directed mutagenesis. Our findings support the proposal that OxaD catalyzes two subsequent oxygenation reactions of the indole nitrogen of roquefortine C, yielding roquefortine L, with the involvement of an *N*-hydroxyl intermediate.^6^ Spectroscopic and mass spectrometry analyses suggest that the *N*-hydroxyl intermediate remains bound after the first oxidation, which is consistent with a sequential hydroxylation mechanism. Steady-state kinetic data indicate that the enzyme does not exhibit significant specificity between NADPH or NADH and displays high catalytic efficiency with roquefortine C. We observed that the slowest step of the reaction is the dehydration of the flavin, suggesting this is a rate-contributing step. Through structural modeling of OxaD and bioinformatic analysis, we identified significant structural and sequence conservation between OxaD and flavin-dependent epoxidases and a *N*-hydroxylase. Notably, an active site aspartic acid residue is conserved among these enzymes, and mutagenesis studies targeting this residue have shown a retention of less than 10% activity or a complete loss of activity.^21−24^ We performed a corresponding mutation in OxaD (Asp63 to Ala), and kinetic characterization confirmed the critical role of this residue in orchestrating a hydrogen bonding network necessary for flavin reduction and product oxygenation.

## Experimental Procedures

### Materials

Bacterial growth media and buffer reagents were purchased from Research Product International (Mt. Prospect, IL). Dimethyl sulfoxide (DMSO) was obtained from Fisher Scientific (Pittsburgh, PA). The genes coding for OxaD (UniProt ID, A0A1B2TT15) and *Thermoanaerobacter brockii* alcohol dehydrogenase (UniProt ID, P14941) cloned into pET28a plasmid were obtained from Genscript Biotech (Piscataway, NJ). *Escherichia coli* BL21 (DE3) and OneShot TOP10 chemically competent cells were obtained from Invitrogen (Carlsbad, CA). Glucose oxidase, 2-propanol-*d*_8_, and molecular weight cutoff centrifugal filters were obtained from Sigma-Aldrich (St. Louis, MO). NAD(P)H was obtained from Research Product International (Mt. Prospect, IL). Roquefortine C was obtained from Cayman Chemical (Ann Arbor, MI). For protein purification, a Cytiva (Marlborough, MA) AKTA start system and HisTrap fast flow 5 mL columns were used. Sonication was carried out using a Fisher Scientific Sonic Dismembrator model 500 (Hampton, NH) with a 3/4 in. probe. The activity following the consumption of oxygen was measured using a Hansatech Clark-type oxygen electrode system (Norfolk, U.K.). Concentration dependent product analysis was performed using a Phenomenex (Torrance, CA) Kinetex C18, 5 μm (2.5 mm × 100 mm) column connected to an HPLC Shimadzu LC-20 series (Kyoto, Japan). Product identification was conducted using a high-resolution LC-MS Shimadzu 9030 equipped with an LC-40B pump (Kyoto, Japan) with a Waters (Milford, MA) C18 BEH, 1.7 μm (2.1 mm × 50 mm) column to resolve peaks of interest. HPLC grade water, acetonitrile, and formic acid were obtained from Fisher Scientific (Pittsburgh, PA). UV–visible spectra under anaerobic conditions were acquired using a Starna Cells (Atascadero, CA) Spectrosil quartz cuvette sealed with a silicone septum. Anaerobic stopped-flow spectrophotometry was performed with an Applied Photophysics SX20 stopped-flow spectrophotometer (Surrey, U.K.) housed inside a COY Laboratories (Grass Lake, MI) glovebox. All gas tanks were supplied by Airgas (Radnor, PA). Oxygen flow was regulated using a Porter Instruments flow meter (Hatfield, PA). Mutagenesis was performed following the manufacturer procedures for Q5 site-directed mutagenesis kit from New England Biolabs (Ipswich, MA). Data fitting analysis was carried out using Kaleidagraph graphing software by Synergy Software (Reading, PA). Spectral deconvolution was conducted using Applied Photophysics ProKIV software (Surrey, U.K.).

### Protein Expression and Purification

The pET28a plasmid containing the OxaD gene was transformed into chemically competent *E. coli* BL21 (DE3) cells. Protein expression was carried out using autoinduction media as described by Fox et al., 2009.^25^ Cells were grown at 37 °C with shaken at 250 rpm until an optical density at 600 nm of 4.0 was reached. The temperature was lowered to 18 °C, and cells were grown overnight. The cells were harvested by centrifugation and stored at −70 °C. For protein purification, the cell pellet was thawed on ice and resuspended in buffer A:25 mM HEPES/Na^+^ buffer, pH 7.6, 300 mM NaCl, 10 mM imidazole, 0.2 mM tris(2-carboxyethyl)phosphine hydrochloride (TCEP-HCl). The solution was supplemented with 1 mM phenylmethylsulfonyl fluoride (PMSF), 25 μg/mL lysozyme, 10 μg/mL DNase I, and 10 μg/mL RNase and was stirred at 4 °C for 20 min. The cell suspension was then sonicated at 70% amplitude for 15 min, with intervals of 5 s on and 10 s off. The lysate was centrifuged at 16 000*g* and 4 °C for 45 min to pellet unlysed cells and insoluble material. The supernatant was diluted 1:2 in buffer A and loaded onto two HisTrap FF, 5 mL columns at 2 mL/min using an AKTA start system. The columns were washed with 5% of buffer B:25 mM HEPES/Na^+^ buffer, pH 7.6, 300 mM NaCl, 300 mM imidazole, 0.2 mM TCEP-HCl. OxaD was eluted using a gradient of 15 mM (5% B) to 300 mM (100% B) imidazole over 10 column volumes at 5 mL/min. The purity of the protein was assessed using SDS–PAGE analysis (samples were resolved using 12% acrylamide/bis acrylamide gels). Fractions with pure protein were pooled and concentrated using a 30 kDa molecular weight cutoff centrifugal filter. The concentrated sample was dialyzed overnight into the storage buffer (25 mM HEPES/Na^+^ buffer, pH 7.6, 200 mM NaCl, 0.3 mM TCEP-HCl, 10% glycerol) at 4 °C. The protein was flash frozen with liquid nitrogen as 10 μL droplets and stored at −70 °C. The bound flavin extinction coefficient was measured by following the procedures previously described.^1,26^ Bradford assay was used to measure protein concentration and determine flavin incorporation.

### Site-Directed Mutagenesis

The OxaD mutant, D63A, was generated using the Q5 site-directed mutagenesis kit from New England Biolabs following the instructions from the manufacturer. The DNA sequence was determined by Sanger sequencing conducted at the Genomic Sequencing Center in the Fralin Life Science Institute at Virginia Tech. OxaD D63A (D63A from hereon) was expressed and purified using the same procedures as those described for OxaD.

### Oxygen Consumption Assay

All oxygen consumption assays were performed using an Oxygraph+ system from Hansatech Instruments (Norfolk, U.K.). Assays were performed in 25 mM HEPES/Na^+^ buffer, pH 7.6, 50 mM NaCl, 1% DMSO (750 μL total volume). OxaD (1 μM) was incubated with roquefortine C at the desired concentration for 2 min prior to the start of the reaction. The reaction was initiated by the addition of NAD(P)H. In experiments where the NAD(P)H concentration was varied (10–500 μM), roquefortine C was held constant at 100 μM. When roquefortine C was varied (2.5 μM to 100 μM), NAD(P)H was kept at 500 μM. For D63A, 1 μM enzyme was used along with 100 μM roquefortine C when NAD(P)H was varied (250–3000 μM), and 2.5 mM NADPH was used when roquefortine C was varied (2.5 μM to 100 μM). Equation 1 was used to determine the turnover number (*k*_cat_) and Michaelis–Menten constant (*K*_M_). To determine reaction coupling, reactions rates in the presence and absence of 1 mg/mL catalase were compared under saturating conditions (OxaD, 1 μM enzyme, 100 μM roquefortine C, 500 μM NADPH; D63A, 1 μM enzyme, 100 μM roquefortine C, 2.5 mM NADPH).
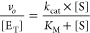
1

### Product Formation Assay

The formation of intermediates and roquefortine L was investigated using RP-HPLC. The assay mixture (500 μL) contained 25 mM HEPES/Na^+^ buffer, pH 7.6, 50 mM NaCl, 1% DMSO, and 0.5 μM OxaD. Roquefortine C (2.5–600 μM) was incubated with the enzyme at room temperature for 2 min before initiating the reaction with the addition of 500 μM NADPH. After 30 s the reaction was quenched by heat inactivation at 80 °C for 3 min. Denatured protein was pelleted by centrifugation at 16 000*g* for 10 min, and the supernatant was transferred to a clean tube. Aliquots (20 μL) of the reaction were injected on the HPLC system equipped with a Phenomenex Kinetex C18, 5 μm (2.5 mm × 100 mm) column equilibrated in 0.1% formic acid (v/v) in water. The samples were eluted with a linear gradient of 0.1% formic acid (v/v) in acetonitrile from 5% to 100% over 35 min at 0.5 mL/min. The concentration of roquefortine C remaining in the reactions was determined by comparing the peak area to those of a standard curve conducted with known concentrations of roquefortine C. The concentration of roquefortine C consumed was used to determine the initial rate (*v*_0_). The data were plotted as a function of roquefortine C concentration and fit to eq 1 to obtain the *k*_cat_ and *K*_M_ values.

### Liquid Chromatography–Mass Spectrometry Analysis

To identify the products of the reaction, a quenched reaction conducted with roquefortine C (100 μM) and NADPH (500 μM) was diluted 1:100 in 90% acetonitrile for liquid chromatography–mass spectrometry analysis (LC–MS). Measurements were performed on a high-resolution LC-MS (Shimadzu 9030) equipped with a LC-40B X3 binary gradient UPLC and autosampler. Solvent A was water containing 0.1% formic acid (v/v) and solvent B was acetonitrile containing 0.1% formic acid (v/v). Samples were injected onto a reversed-phase UPLC Waters C18 BEH column, 1.7 μm (2.1 mm × 50 mm) maintained at 40 °C, with the first minute eluting to waste. The gradient separation began with 5% solvent B (0–2 min) with a linear gradient to 95% B for 10 min. The column was held at 95% B for 2 min before returning to initial conditions at 13 min and was held at the initial conditions (5% B) for an additional 2 min prior to subsequent injections. The flow rate was kept constant at 0.4 mL/min. The compounds were identified based on exact mass, authentic standards, and fragmentation patterns.

### Aerobic and Anaerobic UV–Visible Spectroscopy Analysis

The spectra of different redox states of OxaD and D63A in complex with substrate (or product) were obtained using UV–vis spectroscopy under aerobic and anaerobic conditions. To remove oxygen, solutions were degassed using four cycles of 28 Hg vacuum pressure (4 min each cycle) and 5 psi argon gas (1 min each cycle) with constant stirring. The enzyme solution was made anaerobic using 15 cycles of 28 Hg vacuum pressure (2 s each cycle) and 5 psi argon gas (4 s cycle). To obtain the oxidized enzyme spectra, 10 μM OxaD (or D63A) was diluted in activity buffer (25 mM HEPES/Na^+^ buffer, pH 7.6, 50 mM NaCl, 1% DMSO). The spectra of the oxidized enzymes in complex with roquefortine C were obtained with 10 μM enzyme and 10 μM roquefortine C after 5 min of incubation. The spectrum of reduced enzyme and substrate was obtained by mixing 10 μM OxaD with 10 μM NADPH and 10 μM of roquefortine C in a quartz cuvette sealed with a septum. D63A was reduced with 20 μM of NADPH. The same cuvette was exposed to air, and spectra were acquired 30 s, 1 min, and 2 min after air exposure.

To obtain the spectrum after two oxidations, an anaerobic solution of 10 μM OxaD was incubated for 5 min with 10 μM roquefortine C before the addition of 10 μM NADPH. The sample was kept in a sealed quartz cuvette. After 30 s, the sample was reacted with 10 μM oxygen until the sample was yellow. The spectrum was acquired to ensure the sample was fully oxidized at which point an additional equivalent of NADPH was added (another 10 μM) and the spectrum of the doubly reduced enzyme was acquired. The same cuvette was exposed to the air and spectra were acquired 30 s, 1 min, and 2 min after air exposure.

### Anaerobic Stopped-Flow Spectrophotometry

The reductive and oxidative-half reactions of OxaD and D63A were investigated using a stopped-flow spectrophotometer in an anaerobic chamber. To remove oxygen from the enzyme sample, OxaD (or D63A) was degassed along with buffer solutions as previously described. The stopped-flow was incubated with an anaerobic solution of 0.6 μM glucose oxidase and 100 mM d-glucose in 100 mM sodium acetate, pH 5.0, overnight to remove any oxygen.^27^ Roquefortine C and NAD(P)H were transferred directly to the chamber and resuspended in the anaerobic activity buffer (25 mM HEPES/Na^+^ buffer, pH 7.6, 50 mM NaCl, 1% DMSO). For all stopped-flow experiments, the concentrations listed are after mixing in the instrument. For all experiments where substrate was present, the instrument was blanked with 1 equiv of roquefortine C relative to the enzyme concentration in anaerobic buffer to minimize absorption interference from any unbound substrate or potential products. Flavin reduction was investigated by mixing various concentrations of NAD(P)H (62.5–4000 μM) with 10 μM OxaD, in the absence or presence of 10 μM roquefortine C at room temperature. Kinetic isotope effect experiments were conducted using 4 mM of (*R*)-[4-^2^H]-NADPH and NADPH, which was synthesized alongside the deuterated substrate as a control. Absorbance changes between 200 and 800 nm were recorded. The decrease in absorbance at 446 nm was fit to eq 2 to obtain the observed rate constant of the fast phase (*k*_1_) and of the slow phase (*k*_2_). A_446_ is the absorbance at 446 nm, A is the amplitude of the phase, *t* is time, and C is the final absorbance at 446 nm. The observed rates obtained as a function of NAD(P)H concentration were fit to eq 3, to determine the maximum rate constant for flavin reduction (*k*_red_) and dissociation constant (*K*_D_) for the reducing cosubstrate. Experiments with D63A were carried out using the same procedures as those described for OxaD.
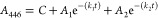
2
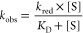
3For the flavin oxidation experiments, reduced OxaD was prepared by mixing 10 μM OxaD with 10 μM NAD(P)H and 10 μM roquefortine C. D63A was reduced using 10 μM enzyme with 20 μM NAD(P)H and 10 μM roquefortine C. Oxygenated buffer was prepared by bubbling 30 cc/min (pressure regulated by a flowmeter) of 100% oxygen into the OxaD activity buffer on an ice bath for 1 h while stirring. The resulting oxygen solution has a concentration of 1.2 mM.^27^ Oxidation of reduced OxaD or D63A was investigated at various concentrations of oxygen (100–600 μM) at room temperature. To obtain lower oxygen concentrations, the saturated oxygen solution was diluted with anaerobic buffer. Absorbance changes at 200–800 nm were recorded. Absorbance changes at 365 nm were fit to eq 4 to account for the absorbance increase and decrease. In eq 4, *A*_365_ is the absorbance values at 365 nm, *A*_1_ and *A*_2_ are the changes in absorbance related to a single phase, *k*_1_ is the rate of absorbance increase, and *k*_2_ is the rate of absorbance decrease. Absorbance increases at 446 nm were fit to eq 5, where A_446_ is the absorbance values at 446 nm, *k*_obs_ is the observed rate and *A*_Δ_ is the change in absorbance. In both eq 4 and eq 5, *A*_0_ is the initial absorbance and *t* is time. The bimolecular rate constant was determined using a linear equation. All data fitting was carried out with KaleidaGraph. Spectral deconvolution was carried out using a three-step model that accounted for the reduced enzyme–substrate complex, the enzyme-intermediate complex, and the final oxidized enzyme.
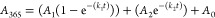
4
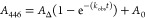
5

### Synthesis of (*R*)-[4-^2^H]-NADPH

NADPH and (*R*)-[4-^2^H]-NADPH were synthesized following the method of Jeong et al., 1994 with some modifications.^28^ 5.5 mM NADP^+^, 1 M 2-propanol-*d*_8_ (for (*R*)-[4-^2^H]-NADPH synthesis) or 1 M 2-propanol (for NADPH synthesis), and 2.5 μM of *T. brockii* alcohol dehydrogenase were added to 25 mM Tris-HCl, pH 9.0. *T. brockii* alcohol dehydrogenase was expressed in *E. coli* BL21 (DE3) and was purified using the same procedures as those described for OxaD with different buffers. Buffer A was 25 mM Tris-HCl, pH 7.3, 300 mM NaCl, 5 mM imidazole, 0.1 mM TCEP; Buffer B was 25 mM Tris-HCl, pH 7.3, 300 mM NaCl, 300 mM imidazole, 0.1 mM TCEP. The protein was stored in 25 mM Tris-HCl, pH 7.3, 300 mM NaCl, 0.2 mM ZnSO_4_, 1 mM TCEP at −70 °C. The reaction was allowed to proceed for 30 min at 40 °C and was stirred at 125 rpm in a benchtop incubator until the A_260_/A_340_ ratio reached a value of ≤2.8. The solutions were filtered with a 3 kDa Amicon molecular weight cutoff filter to remove the enzyme. The filtrate was lyophilized to afford the products as light-yellowish powder. The product was stored at −70 °C and was resuspended in 25 mM HEPES/Na^+^ buffer, pH 7.6, 50 mM NaCl, 1% DMSO for usage.

### Determination of the *K*_D_ of Roquefortine C

The binding of roquefortine C to oxidized OxaD and D63A was monitored by recording the absorbance changes at 365 nm compared to the free oxidized enzyme spectrum using an Agilent 8453 diode array spectrophotometer. These methods were adapted from Abdelwahab et al., 2016.^29^ The buffer used was 25 mM HEPES/Na^+^ buffer, pH 7.6, 50 mM NaCl, 1% DMSO. The instrument was blanked with each concentration of roquefortine C prior to recording the enzyme–substrate complex spectra. Each solution was 200 μL and contained 15 μM of enzyme, and various roquefortine C concentrations (0–40 μM). Prior to recording the spectra, the enzyme was incubated on ice for 5 min with the substrate and the spectra were measured immediately following the incubation. The changes in absorbance at 365 nm were plotted as a function of roquefortine C concentration and were fit using eq 6, where *A* represents the maximum change in absorbance and *L* represents the ligand concentration.
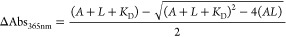
6

### Bioinformatic and Structural Analysis

OxaD homologs were identified using the protein basic local alignment search tool (BLAST) on NCBI.^30,31^ The structural model of OxaD was predicted using the ColabFold server which employs AlphaFold2 and RoseTTAFold.^32,33^ AlphaFold2 includes a confidence score matrix called predicted local distance difference test (pLDDT) that was used for structural validation along with QMEAN calculations by Swiss Model.^34,35^ Structural alignments were conducted using the protein structure comparison service PDBeFold at European Bioinformatics Institute.^36^ Visualization of all protein structures were done with PyMOL by Schrödinger.^37^

## Results and Discussion

### Protein Expression and Purification

Recombinant OxaD was expressed as a fusion with a 6x-His tag using autoinduction in *E. coli* BL21 (DE3) cells and purified using Ni^2+^-NTA chromatography. OxaD was isolated with bound FAD (84% incorporation) at a yield of 9.3 mg of protein per gram of cell (Figure S1). The flavin spectrum of OxaD displays absorbance maxima at 380 and 446 nm, which are characteristic of oxidized FAD (Figure S1). The extinction coefficient of OxaD was determined to be 13.9 mM^–1^·cm^–1^ at 446 nm. Additionally, the spectrum of OxaD features a broad long wavelength absorption band between 500 and 650 nm (Figure S1). Incubation with 10 mM EDTA did not change the spectrum, suggesting this band does not originate from a metal-dependent interaction. We hypothesize that this band could be the result of a charge-transfer complex between the oxidized flavin and a residue in the active site.

### Steady-State Kinetic Analysis

**Figure 1:**
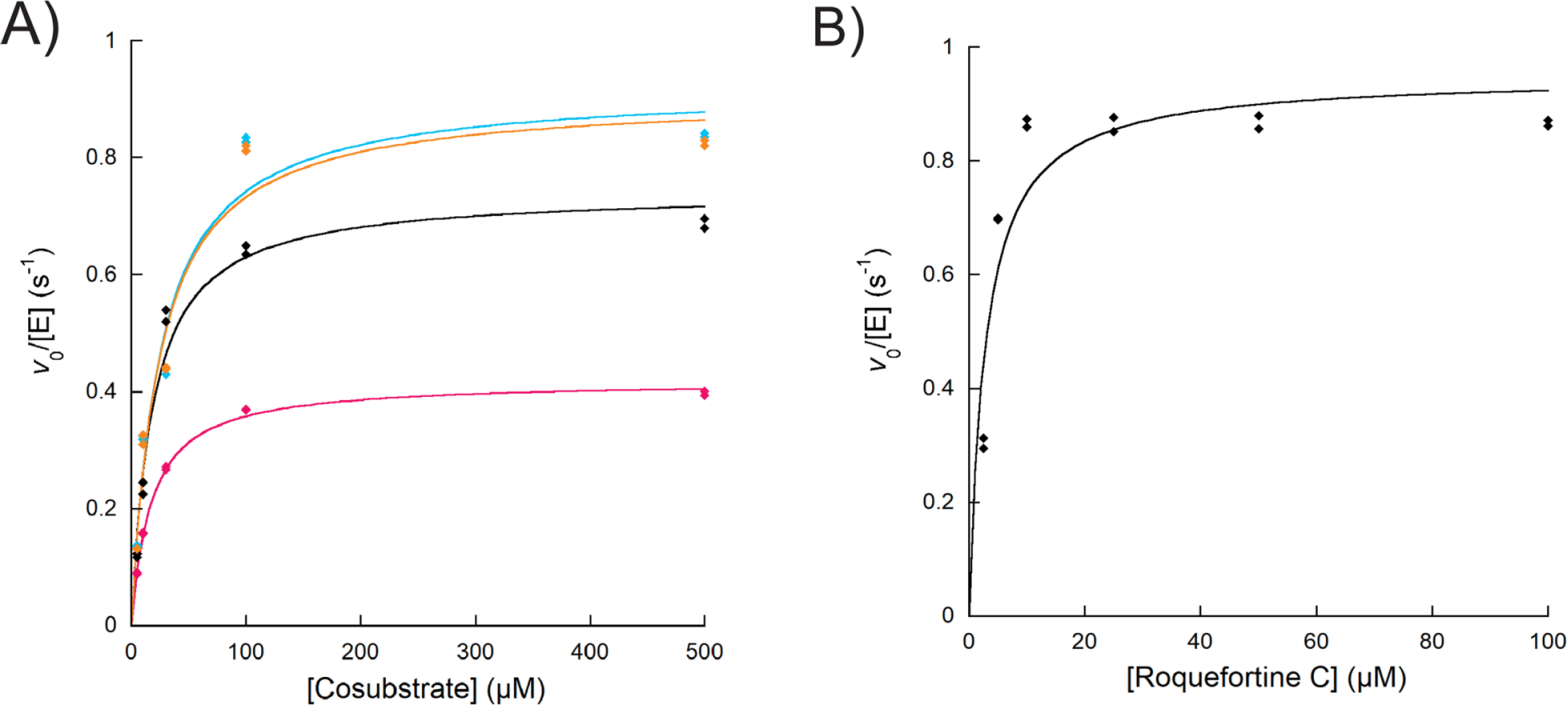
Steady-state kinetics of OxaD using the oxygen consumption assay. (A) Initial velocities at different concentrations of NADPH (blue), synthesized NADPH (orange), (*R*)-[4-^2^H]-NADPH (pink), and NADH (black) Roquefortine C was held constant at 100 μM, and all cosubstrates were tested between 10 and 500 μM. (B) Initial velocities as a function of roquefortine C between 2.5 and 100 μM. NADPH was held constant at 500 μM. The data in both panels were fit to eq 1. In all experiments, the enzyme concentration was 1 μM.

Steady-state kinetic experiments were performed using oxygen consumption and product formation assays. OxaD does not exhibit a significant preference for NADPH or NADH as indicated by similar catalytic efficiencies (Table 1; Figure 1A). When varying roquefortine C, the experiment gave a *k*_cat_/*K*_M_ of 3.1 × 10^5^ ± 1.0 × 10^5^ M^–1^·s^–1^ (Table 1; Figure 1B). Previous kinetic characterization of OxaD reported a lower *k*_cat_ (0.017 s^–1^) and *K*_M_ (71 nM) than our data indicated, however, the *k*_cat_/*K*_M_ values are very similar (2.3 × 10^5^ M^–1^·s^–1^).^6^ To determine the stereochemistry of hydride transfer, we measured the kinetic isotope effect with (*R*)-[4-^2^H]-NADPH. These studies showed a kinetic isotope effect on the *k*_cat_ (^D^*k*_cat_) of 2.30 ± 0.58, indicating that hydride transfer proceeds with pro*R* stereochemistry, which is consistent with other reports of FMO.^38−42^

**Table 1:** Steady-Steady Kinetic Parameters for OxaD and D63A^a^

substrate	*k*_cat_ (s^–1^)	*K*_M_ (μM)	*k*_cat_/*K*_M_ (M^–1^·s^–1^)
**OxaD**			
Roquefortine C	0.93 ± 0.07	3.0 ± 1.0	3.1 × 10^5^ ± 1.0 × 10^5^
NADPH	0.93 ± 0.10	24 ± 7.0	3.8 × 10^4^ ± 9.0 × 10^3^
NADH	0.70 ± 0.10	17 ± 3.0	4.3 × 10^4^ ± 7.0 × 10^2^
Synthesized NADPH	0.91 ± 0.04	23 ± 4.0	3.9 × 10^4^ ± 6.0 × 10^3^
(*R*)-[4-^2^H]-NADPH	0.40 ± 0.10	16 ± 1.0	2.5 × 10^4^ ± 2.0 × 10^3^
^D^*k*_cat_ = 2.30 ± 0.58			
**D63A**			
Roquefortine C	2.0 ± 0.10	3.5 ± 0.90	5.7 × 10^5^ ± 1.3 × 10^3^
NADPH	2.2 ± 0.20	600 ± 200	4.0 × 10^3^ ± 7.0 × 10^2^
Synthesized NADPH	2.1 ± 0.20	630 ± 150	3.7 × 10^3^ ± 4.0 × 10^2^
(*R*)-[4-^2^H]-NADPH	0.62 ± 0.04	420 ± 75	1.5 × 10^4^ ± 2.0 × 10^3^
^D^*k*_cat_ = 3.40 ± 0.38			

a Conditions: 25 mM HEPES/Na^+^ buffer, pH 7.6, 50 mM NaCl, 1% DMSO. Roquefortine C was the fixed substrate (100 μM) to obtain values for reducing cosubstrates, and NADPH was fixed for experiments where roquefortine C was varied (500 μM for OxaD and 2500 μM for D63A). Error values were obtained from the data fitting analysis of two independent, except for kinetic isotope effect values. The errors of the kinetic isotope effects were calculated by propagating the errors associated with the *k*_cat_ values.

We aimed to determine how effectively OxaD couples oxygen activation to product formation as opposed to an unproductive uncoupling reaction that produces hydrogen peroxide. To accomplish this, we performed reactions at saturating conditions (500 μM NADPH and 100 μM roquefortine C) in the presence or the absence of roquefortine C and catalase using the oxygen consumption assay. Catalase catalyzes the conversion of hydrogen peroxide to oxygen and water, slowing the oxygen consumption rate if hydrogen peroxide is present and has been demonstrated to not inhibit other FMOs.^43^ We did not observe any changes in the reaction rate in the presence of catalase, indicating that OxaD is 100% coupled.

A product formation assay was employed to determine how many molecules of molecular oxygen are required for catalysis. The product formation assay was carried out by monitoring the consumption of roquefortine C to determine the *k*_cat_ value. A *k*_cat_ of 0.55 ± 0.08 s^–1^ was calculated, which is approximately half the *k*_cat_ value measured using the oxygen consumption assay (Figure S2). This finding is consistent with OxaD consuming two molecules of oxygen to oxidize one molecule of roquefortine C. To determine the identity of reaction products, we performed LC–MS analysis. Three peaks that were unique to the sample containing OxaD were identified as residual roquefortine C, *N*-hydroxy-roquefortine C, and roquefortine L (Figure S3).

**Figure 2:**
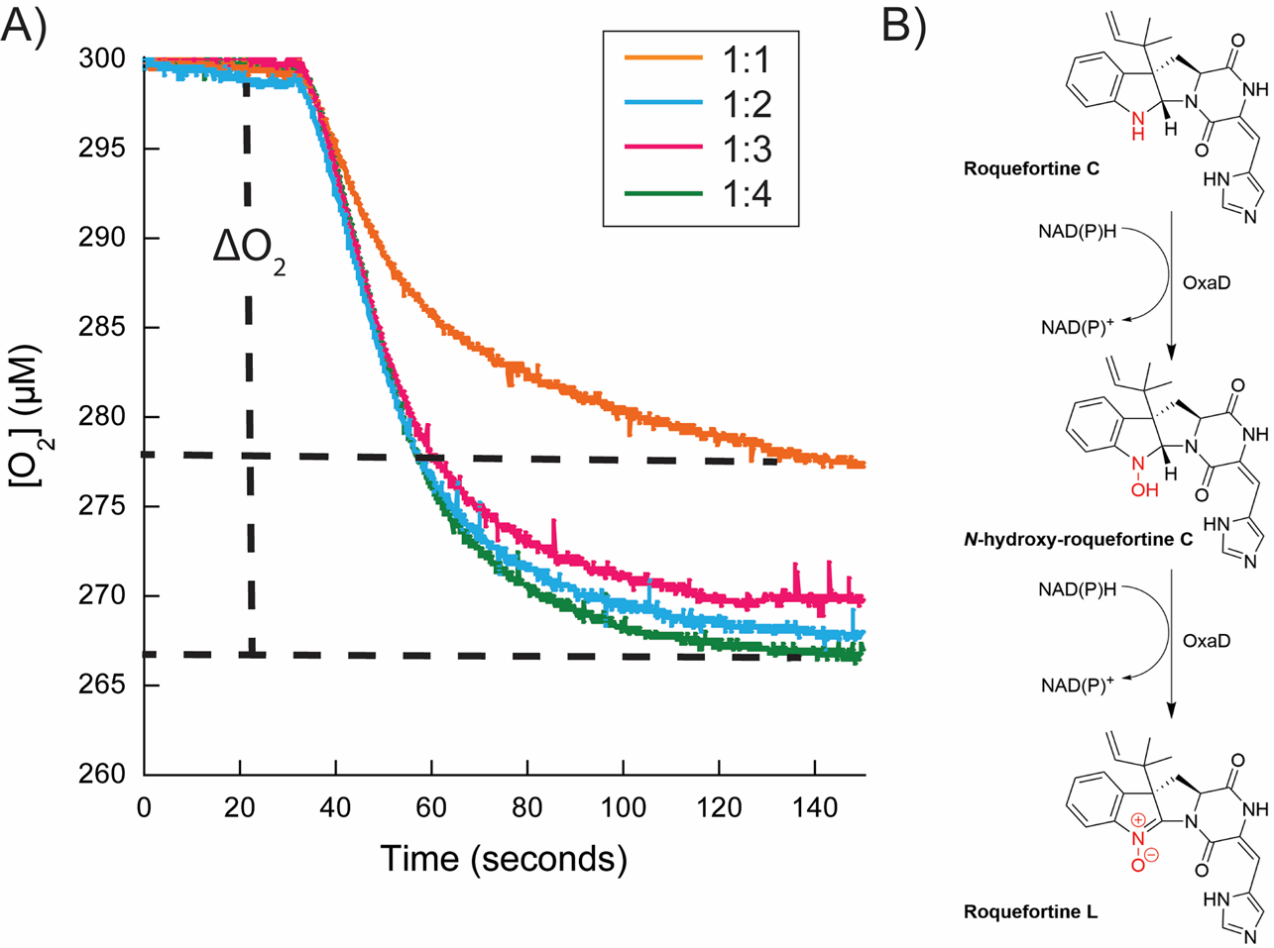
OxaD catalyzes two oxidations of roquefortine C. (A) Representative oxygen consumption traces used to calculate the amount of oxygen consumed. The dashed lines indicate the change in the oxygen concentration from the baseline to the reaction plateau. The concentration of roquefortine C was 15 μM and of OxaD was 3 μM. In a 1:1 ratio of roquefortine C:NADPH, OxaD consumes 18 ± 4 μM of oxygen (orange). A 1:2 ratio gives 34 ± 5 μM of oxygen consumption (blue). The 1:3 ratio resulted in 30 ± 1 μM of oxygen consumption (pink). The 1:4 ratio consumed 33 ± 3 μM (green). The experiment was done in triplicates. (B) The two oxidations catalyzed by OxaD are predicted to be the conversion of roquefortine C to *N*-hydroxy-roquefortine C followed by a second oxidation to form roquefortine L.

Using the oxygen consumption assay, we aimed to probe the multiple oxidations of roquefortine C by OxaD. This was done by performing reactions at a fixed concentration of roquefortine C (15 μM) with various ratios of NADPH. At a ratio of 1:1 of roquefortine C: NADPH, the consumption of oxygen was 18 ± 4 μM (Figure 2). When the ratio was increased to 1:2, 34 ± 5 μM of oxygen was consumed. Increasing the ratio to 1:3 and 1:4 did not change the consumption of oxygen with values of 30 ± 1 and 33 ± 3, respectively (Figure 2). Slight variations in the amount of oxygen that was consumed are due to the background (no NADPH control) that displayed a change of 5 ± 2 μM in the oxygen concentration (not shown). These findings are consistent with the product formation assay, and with prior work which suggested that OxaD undergoes two oxidation cycles to form roquefortine L.^6^

### UV–Visible Spectral Analysis of the Enzyme–Substrate Complex

**Figure 3:**
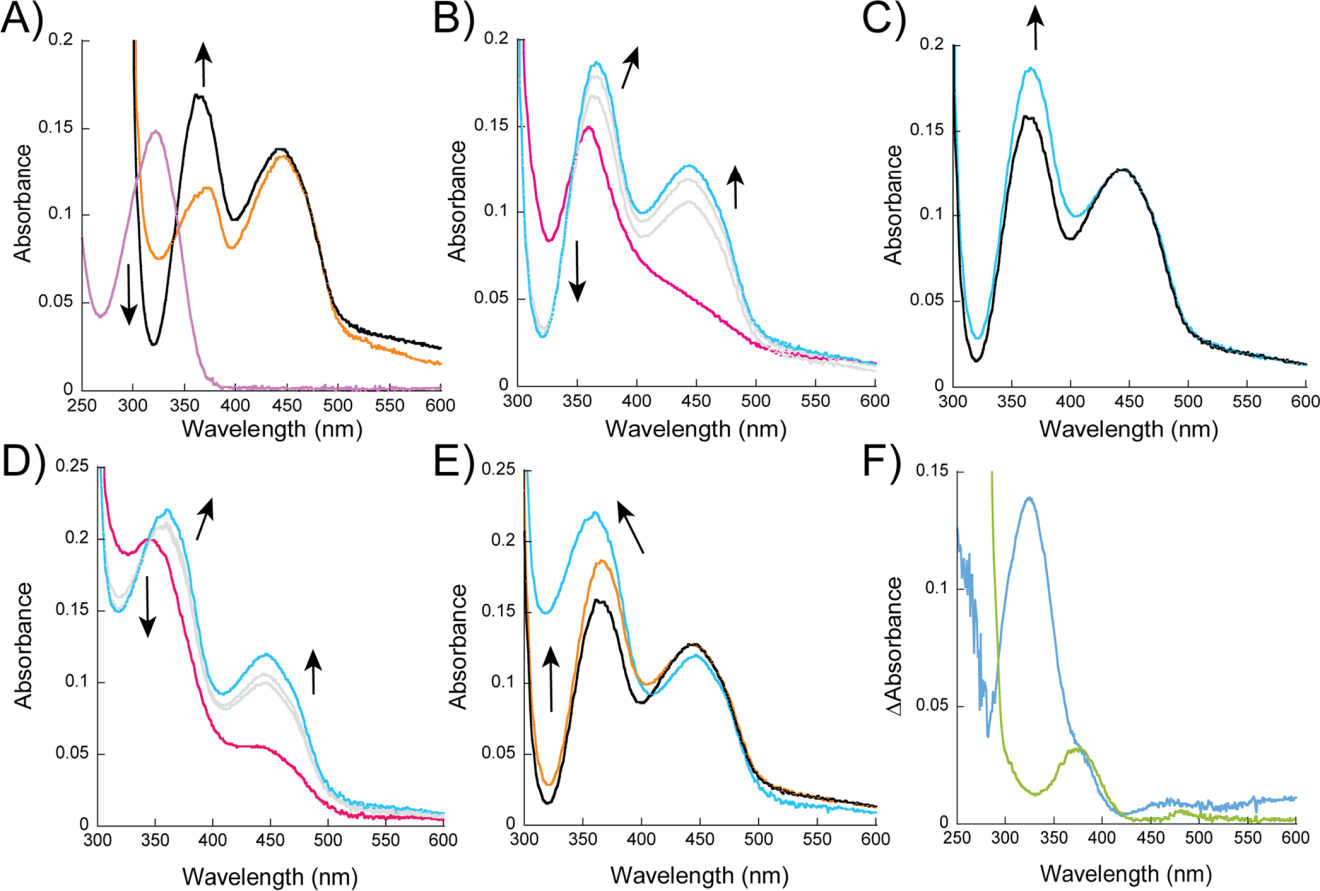
Spectral analysis of the enzyme–substrate/intermediate (or product) complexes. (A) UV–vis spectra of 10 μM roquefortine C (purple) and OxaD (orange) and in complex (black). (B) Spectra of 10 μM reduced OxaD with 10 μM roquefortine C (pink) and 2 min after exposure to air (blue). Gray spectra represent 30 s and 1 min after air exposure. (C) Spectra of oxidized enzyme in complex with roquefortine C (black) and oxidized enzyme after catalysis (blue). (D) Spectra of 10 μM OxaD reduced with 10 μM NADPH after one round of oxidation (pink) and 2 min after exposure to air (blue). Gray spectra represent 30 s and 1 min after air exposure. (E) Spectral comparison of the enzyme–substrate complex without catalysis (black), after one round of oxidation (orange), and after two rounds of oxidation (blue). (F) Subtraction of the enzyme–substrate complex spectrum from the first round of oxidation spectrum (green) and from the second round of oxidation spectrum (blue). In all panels, the arrows indicate the direction of the changes in absorbance.

We found that there are observable UV–visible spectral changes to both roquefortine C and OxaD upon binding and that these changes were different if OxaD was reduced or oxidized. Free roquefortine C displayed a unique absorption peak at 325 nm. OxaD in the oxidized form displays two major peaks at 380 and 446 nm, but when roquefortine C binds to OxaD, a new peak at 365 nm is observed, while the peak at 446 nm remains unchanged (Figure 3A). This complex was stable for 4 h before any decay was observed, which may be the result of a genuine dissociation of the enzyme–substrate complex or protein instability given the long incubation time (not shown). The reduced OxaD spectrum in complex with roquefortine C has a peak at 350 nm, which shifted to 365 nm upon oxidation (Figure 3B). The peak at 365 nm remains higher than the peak at 446 nm for several minutes after oxidation is completed (Figure 3B). There was a small increase in the peak at 365 nm after one oxidation reaction (this corresponds to one oxidation because OxaD, roquefortine C, and NADPH are at 1:1:1 ratio) compared to the enzyme–substrate in the oxidized form (Figure 3C). We hypothesize this might be caused by the complex of OxaD and *N*-hydroxy-roquefortine C, which is formed after one oxidation and may have different active site interactions, causing a small increase in the absorbance.

We aimed to determine the spectral changes after a second oxidation. OxaD was mixed with NADPH:roquefortine C:oxygen at 1:1:1 concentrations as we have described previously. The spectrum was obtained to ensure that OxaD was fully oxidized, and we predict that OxaD is now in complex with *N*-hydroxy-roquefortine C and that an equivalent of NADP^+^ is in the solution resulting from the initial reduction of OxaD. We reduced this sample with another equivalent of NADPH. The enzyme partially reduced (90%), which may suggest that OxaD in complex with *N*-hydroxy-roquefortine C has a lowered affinity for NADPH and would require more than 1 equivalent of NADPH to fully reduce the enzyme (Figure 3D). The enzyme reduced in 15 s, which is the same time frame we observed when roquefortine C is the substrate. After air exposure, the peak shifted from 365 to 355 nm and was broader than the peak observed during the first oxidation (Figure 3E). Subtraction of the enzyme–substrate spectrum from the doubly oxidized spectrum revealed a spectrum that is similar to what has been reported for roquefortine derivatives, and thus we identify this spectrum as roquefortine L (Figure 3F).^44^ Performing the same subtraction for the singly oxidized enzyme only showed the increase at 365 nm that we noted previously (Figure 3F). These results are consistent with *N*-hydroxy-roquefortine C remaining bound to the enzyme after one oxidation and support a sequential hydroxylation mechanism. This conclusion is further supported by the MS analysis of the reaction of OxaD that identified roquefortine C and L and only small amounts of *N*-hydroxy-roquefortine C, corresponding to OxaD concentrations (Figure S3).

### Rapid-Reaction Kinetic Analysis

**Figure 4:**
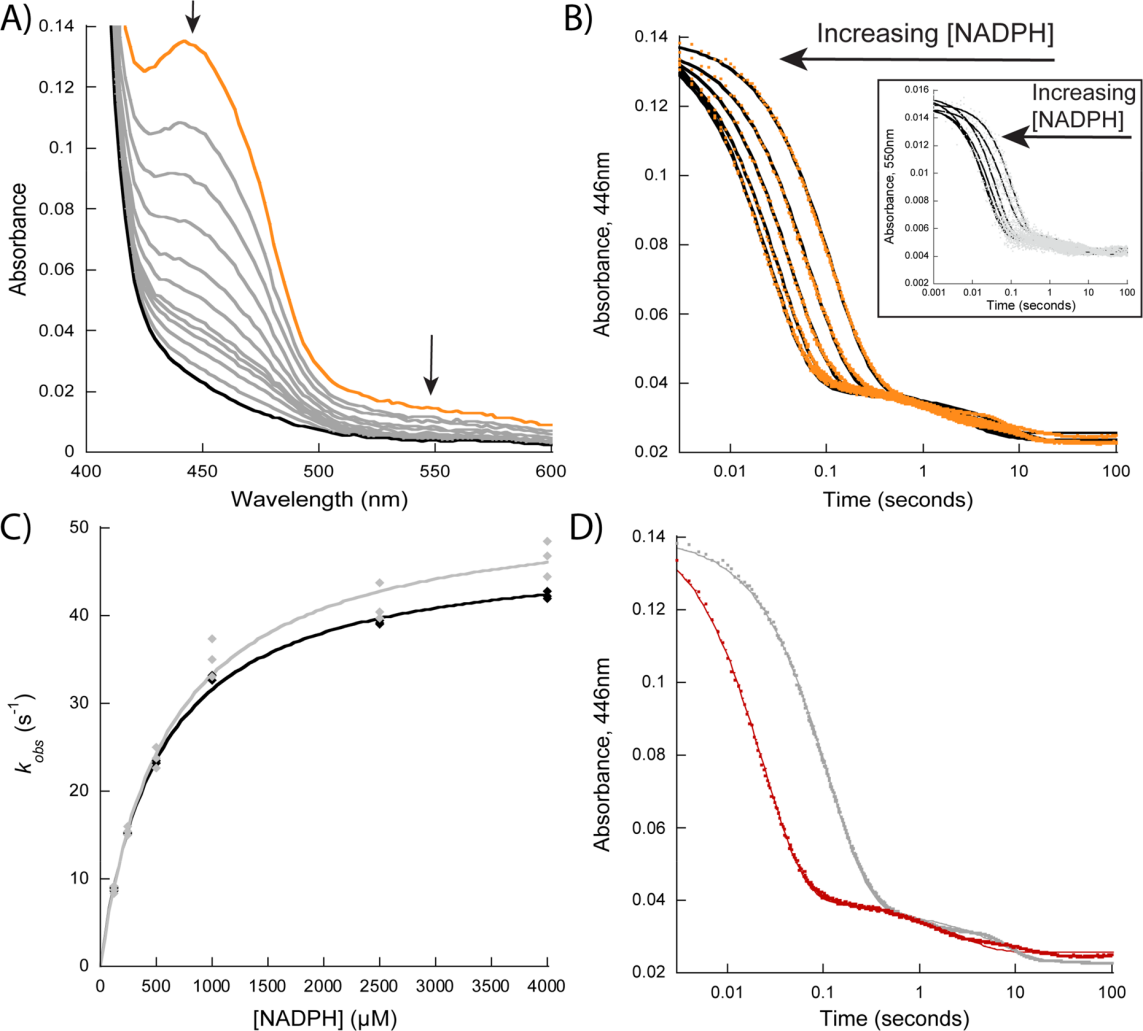
Reductive-half reaction of OxaD. (A) Changes in the flavin spectra upon reaction with 4 mM NADPH over 100 s. The orange line indicates oxidized flavin, and the black indicates reduced flavin. The arrows indicate the direction of the changes in absorbance. (B) Representative reduction traces at 446 nm corresponding to the absorbance bleaching of the oxidized flavin (orange) and at 550 nm (gray, inset). The traces were fit to eq 2 for both wavelengths. (C) Observed rates of reduction at 446 nm (black) and at 550 nm (gray) as a function of NADPH concentration (62.5–4000 μM). The traces were fit to eq 3. (D) Reduction traces at 4 mM NADPH (red) and 4 mM (*R*)-[4-^2^H]-NADPH (gray) were fit to eq 2. OxaD and roquefortine C were both at 10 μM for all experiments.

Stopped-flow spectroscopy under anaerobic conditions was utilized to determine the rapid-reaction kinetic parameters for the reductive and oxidative-half reactions of OxaD. To study the reduction of OxaD we monitored the bleaching of the absorbance of the oxidized flavin at 446 nm in the presence of roquefortine C at equimolar concentrations. Higher concentrations of roquefortine C were not used to prevent further absorbance interference. Because these conditions do not establish first-order conditions, the data presented may not report intrinsic rate constants, thus the rate constants are noted as apparent. OxaD exhibited a biphasic reduction with NADPH, where the fast phase (*k*_red1_) represented ∼95% of the total amplitude change (Figure 4). The fast phase is ∼50-fold faster than the *k*_cat_ value and was dependent on the NADPH concentration with a *K*_D(app)_ value of 0.52 ± 0.03 mM (Table 2).^45,46^ The slow phase was not dependent on the NADPH concentration. Performing the reduction with NADH showed similar results to NADPH, with a ∼ 3-fold increase in the *K*_D(app)_ value, indicating that OxaD binds NADPH with higher affinity than NADH (Figure S4; Table 2). We studied the kinetic isotope effect on the rate of reduction (^D^*k*_red_) with (*R*)-[4-^2^H]-NADPH and determined the ^D^*k*_red_ to be 4.90 ± 0.09 (Figure 4; Table 2). These results further confirm that flavin reduction proceeds with pro*R* stereochemistry and that hydride transfer is the rate-contributing step in reductive half-reaction, which is consistent with other reports on related enzymes.^38−42^ We note that the ^D^*k*_cat_ ∼ 2.1-fold is lower than the ^D^*k*_red_, which indicates that hydride transfer is only partially rate-contributing on the overall catalytic cycle (Tables 1 and 2). The slow phase was not isotope sensitive and likely results from enzyme that was damaged during degassing.

**Table 2:** Rapid-Reaction Kinetic Parameters for the Reductive-Half Reaction^a^

condition	*k*_red1_ (s^–1^)	*k*_red2_ (s^–1^)	*K*_D(app)_ (mM)	*k*_red1_/*K*_D(app)_ (M^–1^·s^–1^)
**OxaD**				
No roquefortine C	0.40 ± 0.10	ND	≥4	NA
NADPH	48 ± 0.80	0.39 ± 0.01	0.52 ± 0.03	9.0 × 10^4^ ± 2.0 × 10^3^
NADH	43 ± 0.30	0.34 ± 0.01	1.6 ± 0.03	3.8 × 10^4^ ± 9.0 × 10^3^
Synthesized NADPH	45 ± 0.70	0.32 ± 0.02	NA	NA
(*R*)-[4-^2^H]-NADPH	9.1 ± 0.10	0.24 ± 0.05	NA	NA
^D^*k*_red_ = 4.90 ± 0.09				
**D63A**				
No roquefortine C	0.17 ± 0.01	N.D.	≥4	NA
NADPH	9.0 ± 0.20	0.18 ± 0.01	1.2 ± 0.30	7.2 × 10^3^ ± 1.4 × 10^3^
Synthesized NADPH	8.6 ± 0.14	0.20 ± 0.04	NA	NA
(*R*)-[4-^2^H]-NADPH	2.4 ± 0.08	0.17 ± 0.01	NA	NA
^D^*k*_red_ = 3.60 ± 0.13				

a Conditions: 25 mM HEPES, pH 7.6, 50 mM NaCl, 1% DMSO. All experiments contained 10 μM roquefortine C, unless otherwise indicated. NAD(P)H concentrations tested were 0.0625–4 mM. Error values were obtained from the data fitting analysis except for kinetic isotope effect values. The errors of the kinetic isotope effects were calculated by propagating the errors associated with observed rate of reduction from three independent experiments.

OxaD displayed a 120-fold decrease in the *k*_red_ when roquefortine C was not present and the *K*_D(app)_ increased significantly as it was not possible to saturate even at 4 mM NADPH (Table 2). Previous work on the class A prototype, para-hydroxybenzoate hydroxylase (PHBH) demonstrates a 10^5^ fold increase in the *k*_red_ in the presence of substrate with no change on the dissociation constant for NADPH.^47^ An earlier work conducted with PHBH reported a similar increase in the *k*_red_ value, but also reported a 9-fold increase in the *K*_D(app)_ for the reducing cosubstrate in the absence of substrate, suggesting that NADPH preferentially binds to the enzyme–substrate complex, which is in agreement with our data for OxaD.^48^ These findings are consistent with the “cautious” mechanism of flavin reduction in which substrate binding triggers the flavin to move from the “in” position to the “out” position where it is exposed to the solvent and NADPH, facilitating hydride transfer.^49−52^ Also of note, in each experiment, there was a decrease in a broad band between 500 and 650 nm, whose rate of decrease matched the flavin reduction rate and we propose that it could be a charge-transfer complex between the oxidized flavin and a residue in the active site, which is lost as the flavin is reduced (Figure 4).

**Figure 5:**
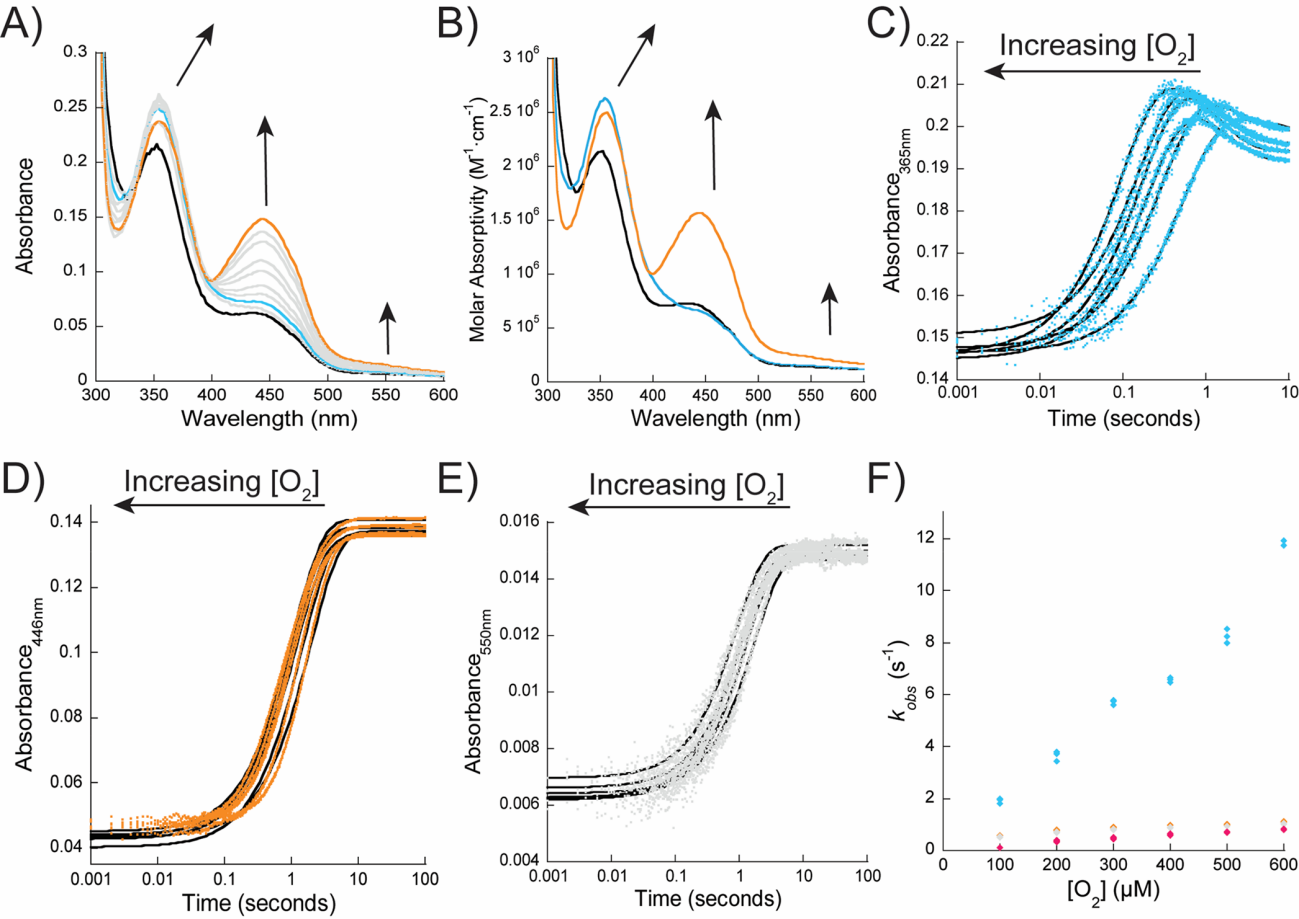
Oxidative-half reaction. (A) Changes in the reduced enzyme-roquefortine C spectra upon reaction with 600 μM O_2_ over 100 s. The reduced enzyme is indicated in black; the enzyme–intermediate complex is shown in blue, and the final oxidation spectra is in in orange. (B) Deconvolution of the spectra from panel A. Spectra were identified to represent the reduced enzyme (black), enzyme–intermediate complex (blue), and the oxidized flavin (orange). (C) Representative rapid-reaction traces (blue) at 365 nm fit to eq 4. These traces represent the formation of an enzyme–intermediate complex. (D) Rapid-reaction traces at 446 nm (orange) fit to eq 5. These traces represent the formation of fully oxidized flavin. (E) Representative traces at 550 nm (gray) were fit to eq 5. In panels C–E, the data fitting is shown in black. (F) Determination of bimolecular rate constant for the formation of the enzyme–intermediate (product) complex (blue), the rate of decrease at 365 nm (pink), the rates of flavin oxidation (orange) and rate of increase at 550 nm (gray) as a function oxygen concentration (100–600 μM). OxaD and roquefortine C were at 10 μM.

We monitored the oxidative-half reaction by measuring absorbance changes upon reaction of the reduced enzyme with oxygen in the presence of roquefortine C using anaerobic stopped-flow spectroscopy. We anticipated that OxaD forms a C4a-hydroperoxyflavin, which would serve as the oxygenating species and is consistent with the mechanisms of other class A FMOs (Figure S3).^51^ The C4a-hydroperoxyflavin has a characteristic absorbance peak at ∼370–390 nm with little change to absorbance in the ∼450 nm region compared to the reduced flavin (Figure 5).^53,54^ Upon reaction of the reduced enzyme-roquefortine C complex with oxygen, we observed the rapid formation of a peak at ∼365 nm that changes very little at ∼450 nm, suggesting this peak represents an intermediate of the catalytic cycle that is formed prior to the reoxidation of the flavin (Figure 5). However, the absorbance increases at ∼365 nm were minor (∼0.05), suggesting that if this spectrum is that of the C4a-hydroperoxyflavin in complex with the substrate, the intermediate would have a low extinction coefficient (Figure 5). Another possibility is that this peak represents a combination of the C4a-hydroperoxyflavin in complex with the substrate and the C4a-hydroxyflavin in complex with the product, which forms after substrate hydroxylation. The rate of increase at 365 nm (*k*_oxy_) was linearly dependent on the oxygen concentration, indicating that the enzyme does not complex with oxygen (Table S1; Figure 5F).

Following substrate hydroxylation, the C4a-hydroxyflavin forms and must be dehydrated to the oxidized form, resetting the enzyme for subsequent catalytic cycles.^51^ The rate constant for flavin dehydration (*k*_OH_; monitored at 446 nm) does not increase as a function of oxygen concentration (Table S1; Figure 5). Of note, *k*_OH_ at atmospheric oxygen concentrations was almost identical to the *k*_cat_ value (*k*_OH_ at 300 μM O_2_ = 0.89 ± 0.06 s^–1^), which is consistent with flavin dehydration being a major rate-contributing step in the catalytic cycle. We observed that as the flavin was oxidizing, there was a small decrease in the absorbance at ∼365 nm (∼0.01) and that this peak does not shift toward the expected wavelength for the flavin of OxaD in the absence of substrate (∼380 nm), indicating that roquefortine derived products were not released (Figures 3 and 5). Fitting of the decrease at 365 nm gave rates that were slightly slower than those of the flavin dehydration rate and were also not dependent on the oxygen concentration (Figure 5). At atmospheric oxygen concentrations (300 μM), the rate of the decrease at 365 nm was 0.65 ± 0.06 s^–1^. We hypothesize that this decrease is related to minor active site rearrangements that are necessary to reset the protein for another round of catalysis and to keep *N*-hydroxy-roquefortine C bound to the enzyme. Additionally, we did observe an increase in a broad band between 500 and 650 nm upon oxidation, with the rates of increase closely resembling the rates of flavin dehydration (Figure 5). This suggests that changes in this region could be related to the interaction between an active site residue and the oxidized flavin.

### Structural Analysis and Site-Directed Mutagenesis

Attempts to crystallize OxaD were not successful. To gain structural information, we generated a model of OxaD using AlphaFold2.^32^ The predicted structure of OxaD resembles that of other class A enzymes as it contains a Rossmann-fold that can bind the adenine nucleotide of FAD and also features a glutathione reductase fold (Figure S5A).^4^ More specifically, the model shares structural similarity with class A epoxidases, FqzB, PhqK, and CtdE, and a *N*-hydroxylase, PaxA (RMSD = 1.78, 1.27, 1.01, and 2.03 Å, respectively) (Figure S5B).^21−24^ Prior studies investigated variant forms of those enzymes and demonstrated that two arginine residues and an aspartic acid were essential for catalysis (Figure S6).^21−24^ These residues are conserved in OxaD and correspond to D63, R125, and R204 (Figure S6). We analyzed the structure of CtdE to develop a hypothesis of the potential role of these residues in catalysis, because the structure was solved with the flavin “in” (positioning for substrate oxygenation) and the flavin “out” (positioning for flavin reduction).^22^ This analysis suggested that interactions between D60 of CtdE (D63 in OxaD) and both arginine residues rearrange when the flavin moves between the “in” and “out” positions (Figure S7). We hypothesized that D60 acts as a “gate-keeper” for flavin motion through rearrangement of hydrogen bonding interactions with R122 (R125 in OxaD) and R200 (R204 in OxaD) and mutated the residue to alanine to test this hypothesis.

### Steady-State Kinetic Analysis of D63A

D63A was recombinantly expressed and purified with very similar results to OxaD. To assess the ability of D63A to generate product, we repeated the product formation assay. We did not observe the formation of any products, which is consistent with previous works (Figure S8). These findings do not rule out NADPH oxidase activity, and we utilized the oxygen consumption assay to determine if D63A was acting as an oxidase. Steady-state kinetic analysis following oxygen consumption of D63A gave a similar *K*_M_ value for roquefortine C as observed with OxaD but displayed a ∼2-fold increase in the *k*_cat_ value compared to OxaD (Figure S9; Table 1). The *K*_M_ value for NADPH also increased by 25-fold (Figure S9; Table 1). We hypothesized that the increase in the *k*_cat_ and *K*_M_ values with NADPH occurred because D63A is acting solely as an oxidase as opposed to OxaD, which catalyzes two successive oxygenation reactions (Scheme S1). To support this hypothesis, we performed reactions at saturating concentrations (2.5 mM NADPH and 100 μM roquefortine C) in the presence and absence of roquefortine C and catalase. Performing reactions with catalase afforded reaction rates that were similar as those in the absence of substrate (0.18 ± 0.03 s^–1^). These data are consistent with an enzyme that is 100% uncoupled and demonstrate that D63A is an oxidase.

### Kinetic Isotope Effects and Flavin Reduction of D63A

To explore the mechanistic changes observed with D63A, we measured the kinetic isotope effect and performed rapid reaction kinetics experiments. The ^D^*k*_cat_ was 3.40 ± 0.38, which suggests that hydride transfer is now more of a rate-contributing step of the catalytic cycle and proceeds with pro*R* stereochemistry (Figure S9; Table 1). This may suggest that the flavin is not optimally positioned for hydride transfer in the “out” conformation and/or that the elimination of a charged residue lowered the flavin reduction potential. Analysis of the flavin reduction demonstrated a ∼5-fold decrease in the *k*_red_ and a ∼3-fold increase in the *K*_D(app)_ for NADPH compared to OxaD (Figure S10; Table 2). We also observed the same decrease in a broad band between 500 and 650 nm with D63A as with OxaD and also note that the rate of the decrease of this band aligned well with the rate of reduction (Figure S10). This suggests that D63 is not the residue responsible for producing the proposed charge-transfer interaction with the oxidized flavin, resulting in the broad band between 500 and 650 nm. Kinetic isotope effect experiments gave a ^D^*k*_red_ of 3.60 ± 0.13, which is lower than that of OxaD, suggesting that hydride transfer is less rate-contributing in the reductive-half reaction of D63A (Figure S10; Table 2). The *k*_red_ is enhanced by ∼50-fold in the presence of substrate, which is a ∼ 2.4-fold lower enhancement than what was observed with OxaD (Table 2). Together these findings suggest that the movement of the flavin between the “in” and “out” positions and the corresponding active site movements are slowed with D63A. This is supported by a slower reduction and a decreased reliance on the presence of substrate compared to OxaD (Table 2).

### Determination of the *K*_D_ for Roquefortine C

To determine if the mutation affected the affinity for roquefortine C, the apparent *K*_D_ value was calculated using UV–visible spectroscopy (Figure S11). We report the data as apparent *K*_D_ values because we cannot distinguish if the spectral changes we observed when roquefortine C binds to the enzyme (formation of a new peak at 365 nm) are related to a change in the absorption properties of roquefortine C, the flavin, or both. The apparent *K*_D_ value for D63A is 6.4 ± 0.20 μM, which is very close the value calculated for OxaD (6.9 ± 0.30 μM), indicating that substrate binding is not impacted by the mutation. Because the reduction is enhanced by the presence of substrate, and we did not observe a change in the binding affinity of the substrate, these findings further support that a slowed velocity of the flavin and active site movements are responsible for the decrease in the flavin reduction rate.

### UV–Visible Spectral Analysis of the Enzyme–Substrate Complex of D63A

We performed the same UV–visible spectral analysis as OxaD with D63A. We observed that the enzyme–substrate complex when no catalysis occurs absorbs at 365 nm, which was also observed in OxaD (Figure S12). The complex between reduced D63A and roquefortine C was shifted from 365 to 360 nm, which was different than the shift we observed from 365 to 350 nm with OxaD (Figure S12). This could suggest that the binding of roquefortine C to the reduced form of D63A is altered compared to OxaD. After exposing the reduced enzyme–substrate complex to oxygen, we observed immediate broadening of the peak at 365 nm, shifting the peak to 355 nm, and that this peak becomes broader until oxidation is completed (Figure S12). Subtraction of the free enzyme spectrum from the spectrum obtained after oxidation was completed revealed a species very similar to the spectrum of free roquefortine C (Figure S12). This may indicate that roquefortine C is being immediately released from D63A or that it is now bound in a manner which does not alter the spectrum of roquefortine C or the flavin.

We aimed to determine if the changes we observed in spectra after catalysis with D63A were present in the oxidized enzyme that did not undergo catalysis and in the reduced form. To test this, we first incubated 10 μM oxidized enzyme with 10 μM roquefortine C and monitored the spectrum for up to 1 h. After 20 min, we observed that the peak at 365 nm was broader than the initial spectrum and that the peak was shifted to 355 nm (Figure S12). These spectral changes occurred much faster than we observed for OxaD and suggest that D63A either releases or rebinds the substrate in a manner that does not impact the spectrum of the flavin or of roquefortine C within 20 min. We did not observe any other changes after 20 min, indicating that the process we have reported is not reversible within 40 min. To determine if these changes were occurring to the reduced enzyme was reduced, 10 μM D63A was reduced using 20 μM NADPH and 10 μM roquefortine C and the spectrum was monitored for up to 1 h under anaerobic conditions. Remarkably, the reduced enzyme–substrate complex took the full hour to observe any of the previously mentioned spectral changes, but like the oxidized forms, the initial spectrum was not observed again after an additional 40 min of spectral monitoring (Figure S12). These findings suggest that the complex between the reduced D63A and roquefortine C is relatively stable compared to those of the oxidized forms. Our data also supports that once dissociation or rebinding of the substrate into an alternative site does occur, the equilibrium does not favor the initial form of the enzyme–substrate complex. We did not observe any dissociation of the same complexes in OxaD within the same time frames, suggesting any form of OxaD complexes stably with roquefortine C. Of note, release after one oxidation was not observed for OxaD, which is consistent with our proposal that *N*-hydroxy-roquefortine C remains bound to the enzyme (Figure 3).

Other class A FMOs have been proposed to adopt additional conformations than the ones we have already discussed known as the “open” and “closed” conformations.^50,57^ In the “closed” conformation the substrate does not have access to the active site and primarily serves to keep reaction intermediates in the enzyme active site until product is formed.^50^ Studies of PHBH investigated the role of flavin movement in substrate binding by covalently linking the flavin to the protein to restrict the flavin movement.^58^ This demonstrated that movement of the flavin out of the “closed” conformation is essential for substrate binding as the rate of binding was determined to be 10^7^-fold slower in the enzyme where the flavin was covalently attached.^50,56,58^ Previous studies have proposed that the “open” conformation is adopted to allow access of substrates to the enzyme active site.^50,57^ If OxaD does also employ these two conformations, we propose that the equilibrium between the “open” and “closed” states is disrupted with D63A, leading to the release of the substrate from the enzyme active site entirely.

### Oxidation of D63A

We aimed to study the oxidative-half reaction of D63A in greater detail and did this through anaerobic stopped-flow spectroscopy. We observed an absorption peak at ∼360 nm for the reduced enzyme in complex with roquefortine C, which shifted to 365 nm upon oxidation (Figure S13). The rate of absorbance increases at 365 nm matched the rate of flavin oxidation at 446 nm, further supporting that there is no formation of an enzyme-intermediate complex in D63A (Figure S13). This is also supported by spectral deconvolution, which was not able to identify an intermediate that does not contain a large amount of oxidized enzyme (Figure S13). We also observed that the peak at ∼365 nm decreases slowly (∼0.01 s^–1^) after the enzyme is oxidized and that this not dependent on oxygen concentration, however, the amplitude of the decrease does increase as the oxygen concentration increased (Figure S13). The absorbance between ∼300 and 330 nm decreases close to zero after oxidation, and considering the instrument is blanked with roquefortine C (absorbs between 300 and 330 nm), this would suggest that roquefortine C is in the solution and is consistent with our UV–visible spectral analysis, which suggested that roquefortine C is released (Figures S12 and S13). We hypothesize that the release of roquefortine C is not dependent on the oxygen concentration, but rather, more of the enzyme population has released roquefortine C in the time frame that we monitored the oxidative-half reaction (100 s) and because oxidation is faster as the oxygen concentration increases, this results in the larger decrease at 365 nm (Figure S13). Therefore, it seems most likely that D63 has a role in positioning the substrate optimally for hydroxylation and also has a role in retaining the substrate in the active site. Also of note, we observed the same increase in a broad band between 500 and 650 nm as OxaD, which also matched the rate of flavin oxidation. This indicates that the charge-transfer we have associated with this band is not impacted by the mutation (Figure S13). Flavin oxidation (*k*_ox_) for D63A is a rate-contributing step, as the value at atmospheric oxygen concentrations is close to the *k*_cat_ value (*k*_obs_ at 300 μM O_2_ is 2.4 ± 0.20 s^–1^).

The data presented here cannot rule out the possibility that new interactions arose between water molecules and/or among residues in the active site because of the void and loss of charge generated from the mutation of an aspartic acid to alanine. Previous work that mutated the corresponding aspartic acid in class A epoxidases and a *N*-hydroxylase to leucine or asparagine as well as alanine.^21−24^ These studies found that the mutants to leucine or asparagine were unable to hydroxylate the corresponding substrate, as was the case with the mutations to alanine.^21−24^ Considering the findings of previous work and the data presented here, we propose that D63 is part of a larger hydrogen bonding network that must globally rearrange to maintain proper active site architecture for productive catalysis. This proposal is supported by other works on PHBH that demonstrate the importance of charged interactions and proper hydrogen bonding network rearrangement for substrate recognition and subsequent catalysis.^52,59^

## Conclusions

Class A FMOs have long been recognized for their involvement in the degradation of aromatic compounds and natural product biosynthesis.^3,60^ Over the past six decades, significant efforts have been dedicated to characterizing class A FMOs, starting with the discovery of salicylate hydroxylase in 1965.^61^ Among these, PHBH stands as the most extensively studied and is often considered the class A prototype.^60,62^ This work represents the first comprehensive kinetic characterization of a *N*-hydroxylase from class A, with a focus on OxaD. Given the prominence of PHBH in class A studies, we will draw comparisons between the proposed mechanism of OxaD and the well-established mechanism of PHBH.

**Figure 6:**
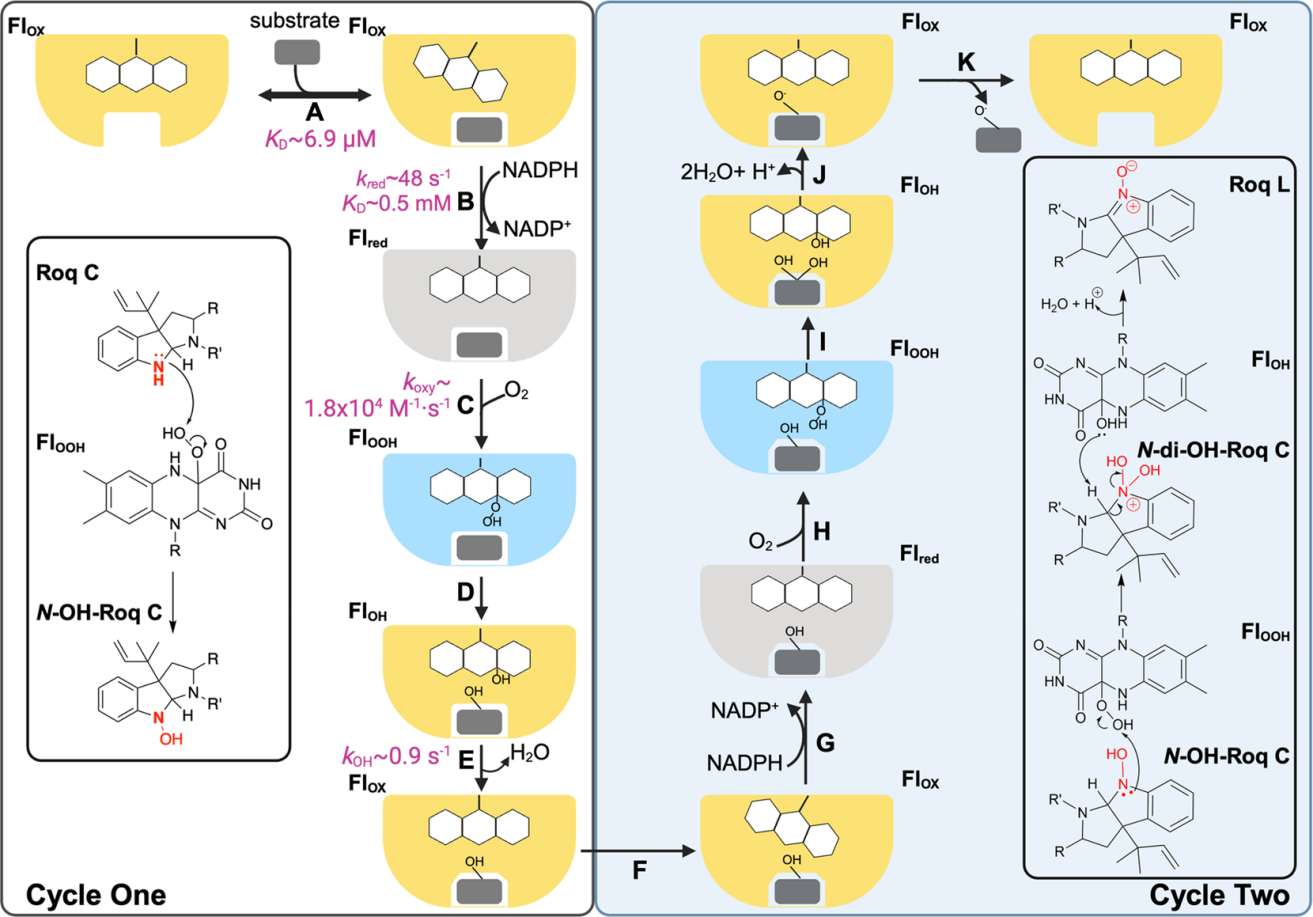
Proposed mechanism of OxaD. The first cycle of the catalysis starts with the binding of roquefortine C to the oxidized flavin in the “in” position (Fl_ox_; step A), which triggers the flavin to move to the “out” position. In the “out” position, the flavin is reduced via hydride transfer from NADPH (Fl_red_; step B) and moves back to the “in” position. The reduced flavin reacts with oxygen to form the C4a-hydroperoxyflavin (Fl_OOH_; step C), which is the oxygenating species. The figure inset depicts the predicted chemical mechanism of the formation of *N*-OH-roquefortine C via nucleophilic attack from the indole nitrogen to the C4a-hydroperoxyflavin. Following product formation, the C4a-hydroxyflavin is formed (Fl_OH;_ step D). The flavin is then dehydrated to the oxidized form, and *N*-OH-roquefortine C is retained in the active site (step E). *N*-OH-roquefortine C triggers the movement of the flavin to the “out” position to kick start the next cycle (step F). The hydride from NADPH is transferred to reduce the flavin a second time, and the flavin moves to the “in” position (step G). Reaction with oxygen will form the second C4a-hydroperoxyflavin, generating *N*-di-OH-roquefortine C through a nucleophilic attack (step H; inset). The C4a-hydroxyflavin would deprotonate the indole to produce a double bond and would release one of the hydroxyl groups as water, generating the oxidized flavin via dehydration (step I; inset). The expected low p*K*_a_ value for this intermediate suggests that it can deprotonate nonenzymatically to form roquefortine L and upon release of roquefortine L, this cycle can restart (step K; inset). In all cases, where the flavin is oxidized the protein is depicted in yellow, where it is reduced it is indicated in gray and where the oxygenating species forms it is depicted in blue. Figure was created with BioRender.com.

Building on the data presented here, we propose a catalytic cycle for OxaD, as depicted in Figure 6. The cycle begins with the binding of roquefortine C to OxaD with the flavin in the oxidized form (Fl_ox_) followed by the rapid reduction, where the pro*R* hydride on the C4 position of NAD(P)H transfers to the N5 position of the flavin isoalloxazine (Figure 6A,B). Notably, hydride transfer constitutes the rate-contributing step in the reductive-half reaction of both OxaD and PHBH (Table 2).^52^ While PHBH exhibits a 10^5^-fold increase in reduction rate in the presence of substrate, roquefortine C binding to OxaD leads to a more modest 120-fold enhancement. This aligns with the “cautious” mechanism utilized by class A FMOs to prevent the wasteful production of hydrogen peroxide.^47,63^ Such increases in reduction rate differ from the “bold” mechanism, where the rate of reduction remains unaffected by the presence of substrate.^39,51,64^ Following the formation of the reduced flavin (Fl_red_), the enzyme reacts with oxygen to form the C4a-hydroperoxyflavin intermediate (Figure 6C). Although our oxidation spectra and kinetic analysis did not conclusively confirm the presence of the C4a-hydroperoxyflavin (Figure 5), our mass spectrometry data identified *N*-hydroxy-roquefortine C as an intermediate in the OxaD reaction, implying the involvement of the C4a-hydroperoxyflavin in the formation of a hydroxylated reaction intermediate (Figure S3). The absence of the C4a-hydroperoxyflavin at room temperature in PHBH has been attributed to the rapid rate of substrate hydroxylation, which may also apply to OxaD.^55,56^ From an observational standpoint, following the reaction with molecular oxygen, flavin dehydration ensues, a step that ultimately limits turnover, mirroring the mechanism of PHBH (Figure 6D,E).^55^

Notably, unlike PHBH, steady-state kinetic analysis revealed that OxaD performs two oxidation reactions to convert roquefortine C into roquefortine L, in agreement with a previous report (Figures 2 and S3).^6^ Previous studies of other multiple-oxidizing FMOs hypothesized that these enzymes might utilize a “release–hydroxylation–recapture” mechanism, wherein the intermediate is released from the active site but remains bound to a “capture” site until another round of reduction occurs.^65^ We propose that the “capture” site does not significantly differ from the original substrate binding site and that the conformational change required to hold onto *N*-hydroxy-roquefortine C would be small. Based on the previous proposal and our spectral data that suggested the presence of a stable complex between *N*-hydroxy-roquefortine C and OxaD, we propose that *N*-hydroxy-roquefortine C remains bound to the enzyme (Figure 6F). We hypothesize that when OxaD complexes with *N*-hydroxy-roquefortine C, the hydrogen-bonding network rearranges (induced by the presence of the hydroxyl group), enabling the flavin to transition to the “out” position for reduction, initiating another oxidation. Following the second oxidation, roquefortine L is released as the final product of the reaction (Figure 6G–K).

Mutagenesis studies on class A epoxidases and a *N*-hydroxylase that share substantial sequence similarity (∼40%) and align well with the structural model of OxaD (RMSD ≤ 2.0 Å) have highlighted the critical roles of two arginine residues (R125 and R204) and an aspartic acid (D63) in catalysis.^21−24^ Previous research suggested that these residues might contribute to flavin binding, flavin reduction, and/or substrate protonation.^21−24^ Our analysis demonstrates that each of these residues participates in a hydrogen bonding network that rearranges to accommodate flavin motion during catalysis (Figure S7). Structural comparisons with PHBH reveal the lack of conservation of these residues, underscoring the presence of a distinct hydrogen bonding network in class A epoxidases and *N*-hydroxylases. Kinetic characterization reveals that D63A follows a similar catalytic cycle to OxaD but fails to generate product. Instead of forming the C4a-hydroxyflavin and product, D63A generates the C4a-hydroperoxyflavin, which decays into H_2_O_2_ and the oxidized flavin (Figure S13). The reported inactivity of D63A in previous studies can be attributed to their reliance on product formation as a measure of activity.^21−24^

In summary, we have provided a comprehensive kinetic characterization of OxaD and D63A. These results expand our knowledge of FMOs involved in natural product biosynthesis that catalyze multiple oxidations. Our data-driven proposal of a detailed catalytic mechanism lays the groundwork for future exploration of OxaD as a candidate for chemoenzymatic synthesis of nitrones. Despite mechanistic similarities with PHBH, OxaD exhibits only moderate structural conservation (RMSD = 2.54 Å) and lacks sequence identity (17%) with PHBH compared to class A epoxidases and *N*-hydroxylases (RMSD ≤ 2.0 Å). This lack of conservation suggests that class A epoxidases and *N*-hydroxylases have evolved to utilize different residues in catalysis and substrate binding compared to well-characterized class A enzymes such as PHBH. Among the epoxidases and *N*-hydroxylases currently under study, OxaD stands as the sole enzyme with a commercially available substrate, facilitating future investigations. Further structural elucidation of OxaD and D63A holds the potential to shed light on roquefortine C binding, multiple oxidation mechanisms, and flavin motion within class A epoxidases and *N*-hydroxylases.

## Supporting Information Available

SDS–PAGE and UV–visible spectra of purified OxaD along with the HPLC and LC–MS analysis of the OxaD reaction; structural model of OxaD with alignment to closely related enzymes and visualization of the active site; determination of the *K*_D_ values of roquefortine C with OxaD and D63A and all kinetic analysis of D63A (PDF)

## Accession Codes

OxaD, UniProt ID: A0A1B2TT15. Alcohol dehydrogenase, UniProt ID: P14941.

## Abbreviations

FMO: flavin-dependent monooxygenase
PHBH: para-hydroxybenzoate hydroxylase
OxaD D63A: D63A
