# Supplementary material for: Mechanism of Nitrone Formation by a Flavin-Dependent Monooxygenase
Source: Biochemistry. 2024 May 23;63(11):1445–59. doi: 10.1021/acs.biochem.3c00656 (PMC11154958; doi:10.1021/acs.biochem.3c00656)
Supplement: Supplementary file 1 — bi3c00656_si_001.pdf [file bi3c00656_si_001.pdf]

Supplemental Information

Mechanism of Nitrone Formation by a Flavin-Dependent Monooxygenase

Sydney B. Johnson[a], Hao Li[a,†], , Hannah Valentino[a,‡], Pablo Sobrado[a,b,*]

[a] Department of Biochemistry and [b] Center of Drug Discovery, Virginia Tech, Blacksburg, VA 24061, United States

*psobrado@vt.edu

Table of Contents

		Page
Figure S1	Purification of OxaD	3
Figure S2	Product formation assay	4
Figure S3	Mass spectrometry analysis of OxaD reaction products	5
Figure S4	Reductive-half reaction results with NADH	6
Figure S5	Structural modeling of OxaD	7
Figure S6	Conservation of residues predicted to play a role in flavin motion in OxaD and class A epoxidases	8
Figure S7	Flavin and active site motion in CtdE	9
Figure S8	HPLC chromatogram of the product formation assay for the D63A	10
Figure S9	Steady-state kinetic analysis of D63A	11
Scheme S1	Schematic representations of the predicted rate-contributing steps of OxaD and D63A	12
Figure S10	Reduction of D63A	13
Figure S11	Binding affinity of roquefortine C to OxaD and D63A	14
Figure S12	Spectral analysis of D63A	15
Figure S13	Oxidative-half reaction results of D63A	16
Table S1	Rapid-reaction kinetic parameters for the oxidative-half reaction	17

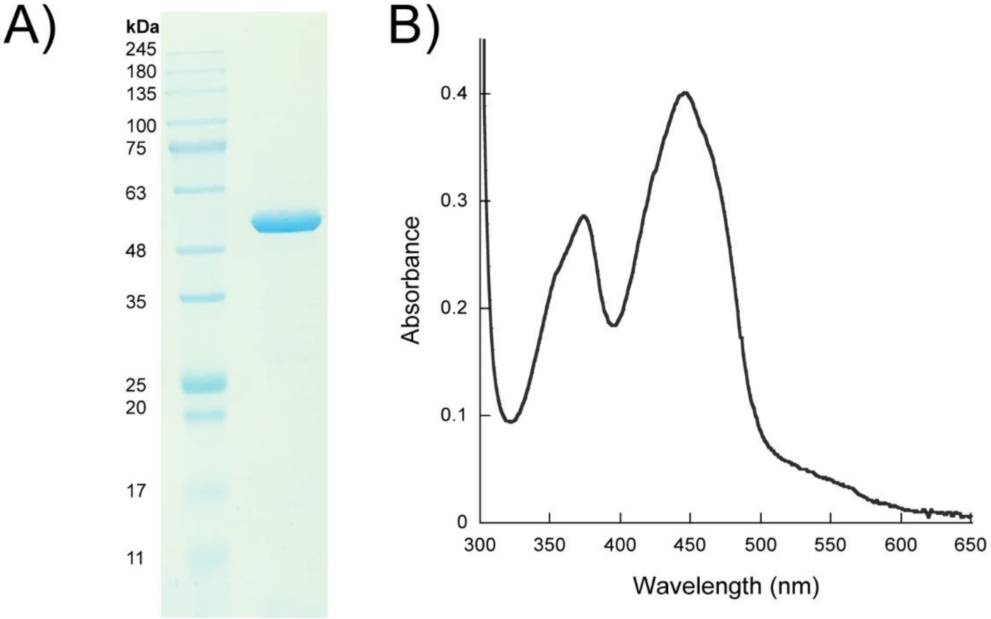

Figure S1. Purification of OxaD. A) SDS-PAGE analysis of the purified OxaD protein shows a band at the predicted molecular weight of 52 kDa. B) The spectrum of OxaD displays peaks at 380 nm and 450 nm, which are consistent with a flavin-bound protein.

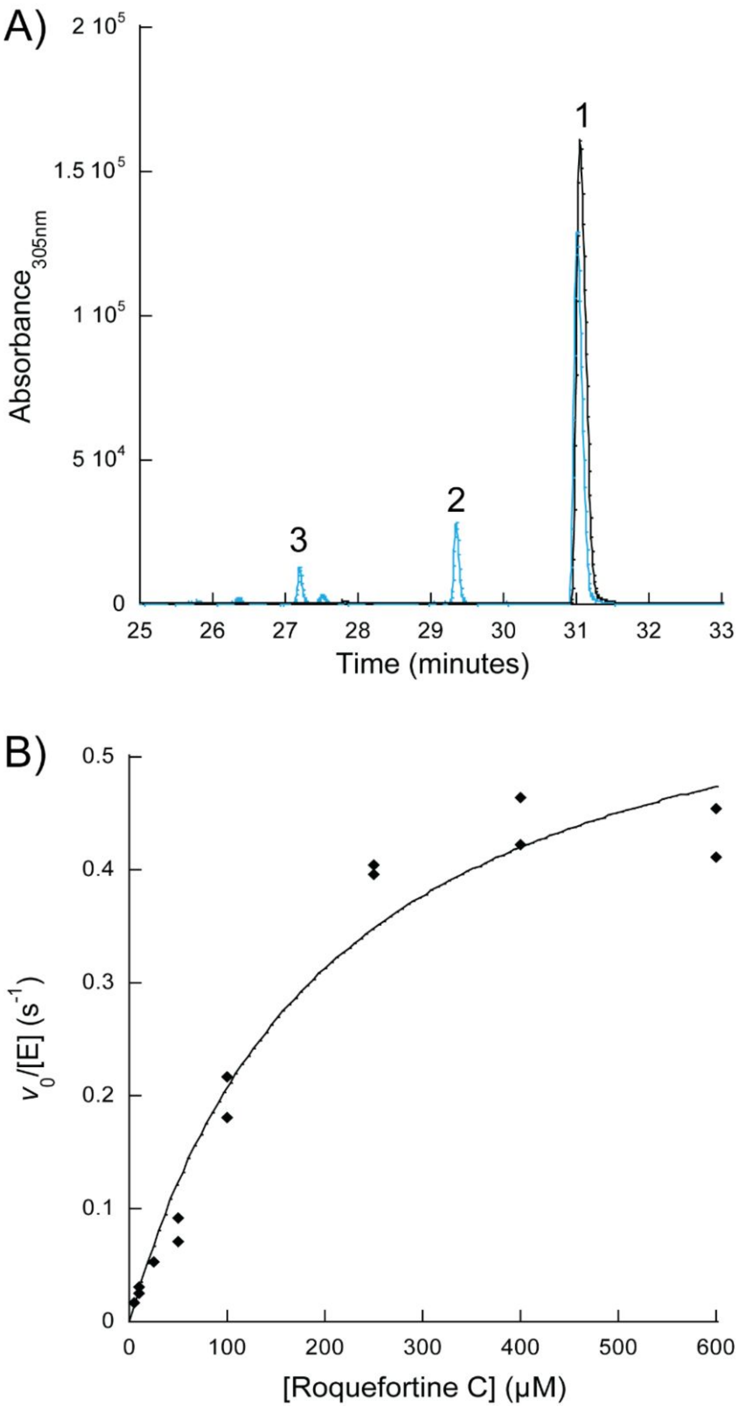

Figure S2. Product formation assay. A) Representative HPLC chromatogram. The blue chromatogram represents an assay sample that contained 100 μM roquefortine C, 500 μM NADPH, and 0.5 μM OxaD. The black chromatogram is the 100 μM roquefortine C standard. Peak 1 was identified as roquefortine C, peak 2 as *N*-hydroxy-roquefortine C, and peak 3 as roquefortine L. B) Initial rates as a function of roquefortine C consumption. The data was fit to Eq. (1). The concentrations of roquefortine C tested were 2.5-600 μM with OxaD at 0.5 μM and NADPH at 500 μM.

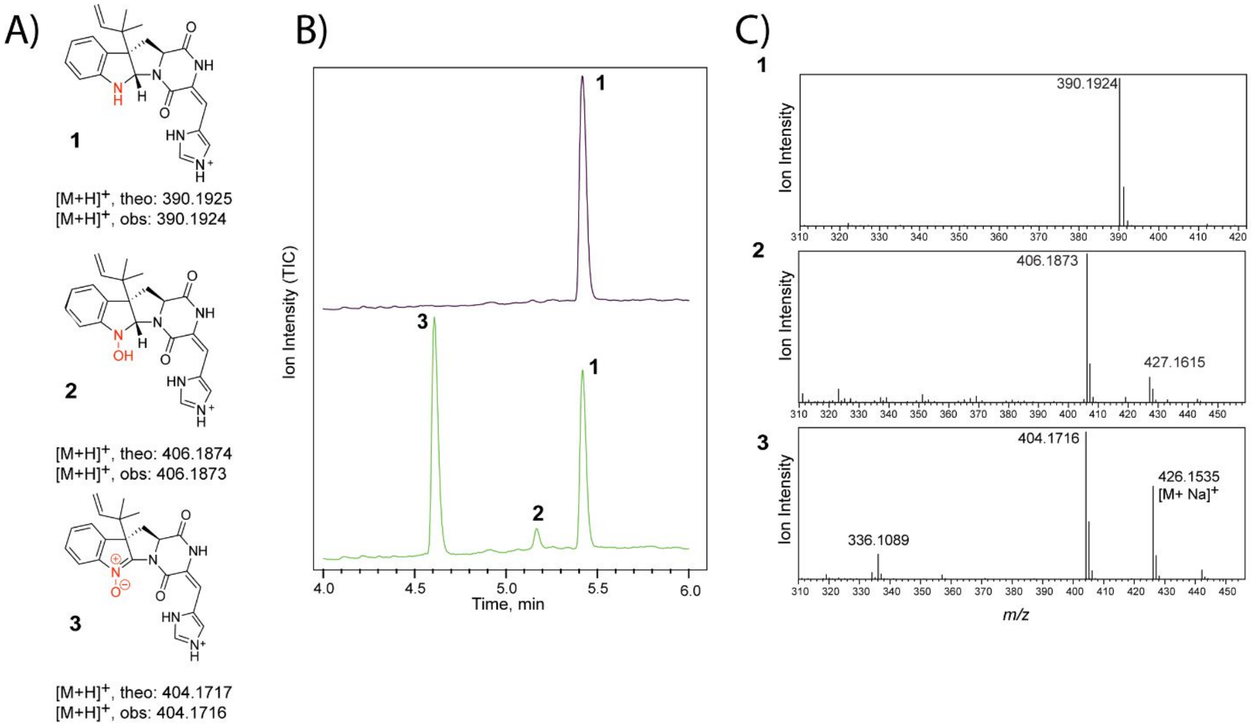

Figure S3. Mass spectrometry analysis of OxaD reaction products. A) Structures of roquefortine C (1), *N*-hydroxy-roquefortine C (2), and roquefortine L (3). Theoretical and observed masses for each compound are listed under the respective structure. B) LC chromatogram displaying the separation of the compounds. The purple chromatogram is the no enzyme control, and the green chromatogram is the reaction with enzyme. C) Mass spectra of each resolved peak. Peak 1 was identified as roquefortine C. Peak 2 was identified as *N*-hydroxy-roquefortine C. Peak 3 was identified as roquefortine L.

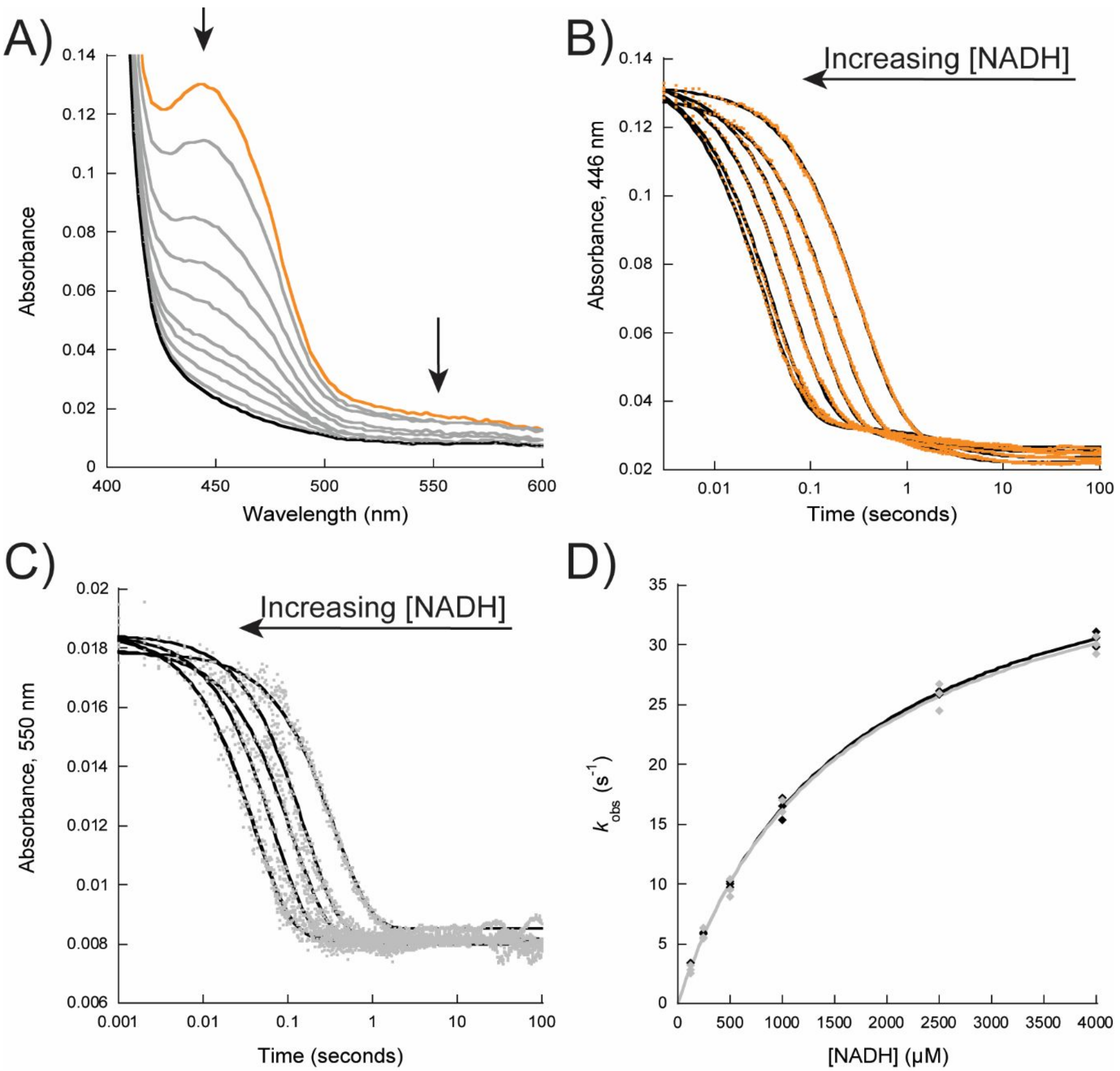

Figure S4. Reductive-half reaction results with NADH. A) Reduction spectra with 4 mM NADH over 100 seconds. The orange line indicates oxidized flavin and the black indicates fully reduced flavin. The direction of the changes in absorbance are indicated by the arrows. B) Representative reduction traces as the NADH concentration increases were fit to Eq (2). C) Experimental traces following the decrease in absorbance 550 nm as a function of NADH concentration (62.5-4000 µM) were fit to Eq. (2). D) Observed rates of reduction as a function of NADH at 446 nm (black) and at 550 nm (gray). The line is a fit to Eq. (3). Roquefortine C and OxaD were at 10 µM.

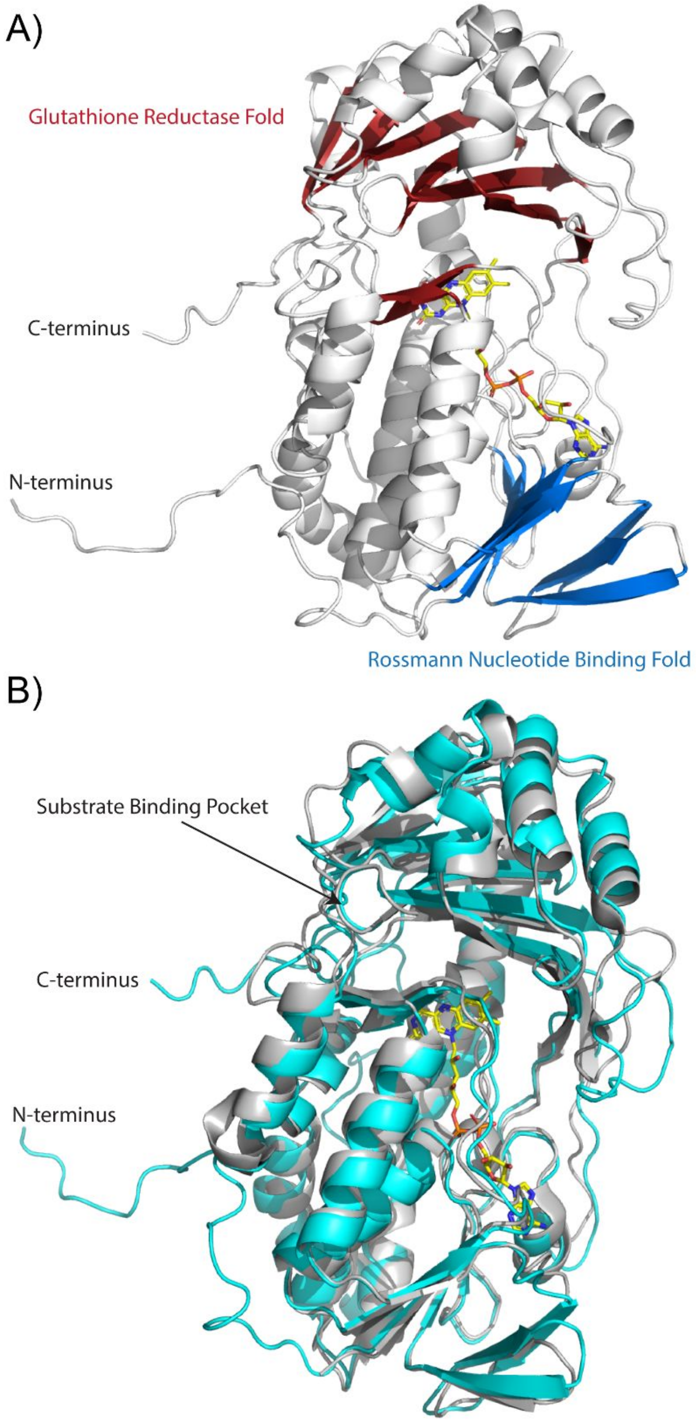

Figure S5. Structural modeling of OxaD. A) Overall structure of OxaD shows glutathione reductase fold (red) and a Rossmann-fold (blue). B) Alignment of OxaD (cyan) to CtdE (PDB ID: 7KPT; gray) has a low RMSD (1.012 Å) and indicates high structural conservation. FAD is shown as sticks and is colored with a yellow carbon backbone in both panels.

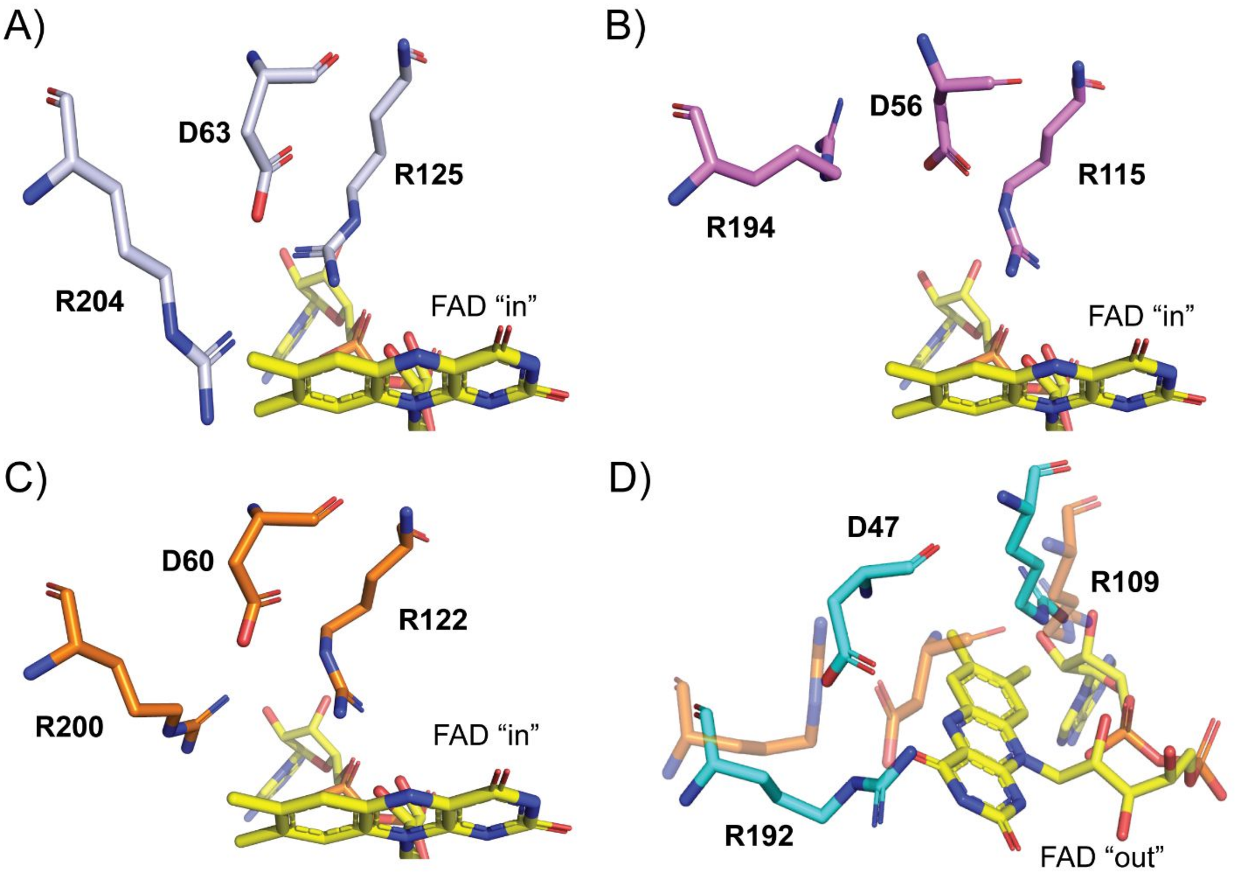

Figure S6. Conservation of residues predicted to play a role in flavin motion in OxaD and class A epoxidases and *N*-hydroxylases. A) Positioning of D63, R125, and R204 in OxaD. Residues are shown in gray. B) View of the residues that align to OxaD’s D63, R125, and R204 in FqzB. The residues are shown in pink. C) The residues that align to D63, R125, and R204 in OxaD are shown in orange in CtdE. D) Flavin “out” position of the equivalent residues to D63, R125, and R204 in PhqK, which does not have the structure solved with the “in” position (blue). The PqhK structure was aligned to the flavin “out” structure of CtdE for comparison (orange; RSMD= 1.88Å). In all panels, the FAD carbon backbone is colored in yellow. PDB IDs: 7CP6 (FqzB), 7KPT (CtdE; flavin “in”), 7KPQ (CtdE; flavin “out”), and 6PVH (PhqK).

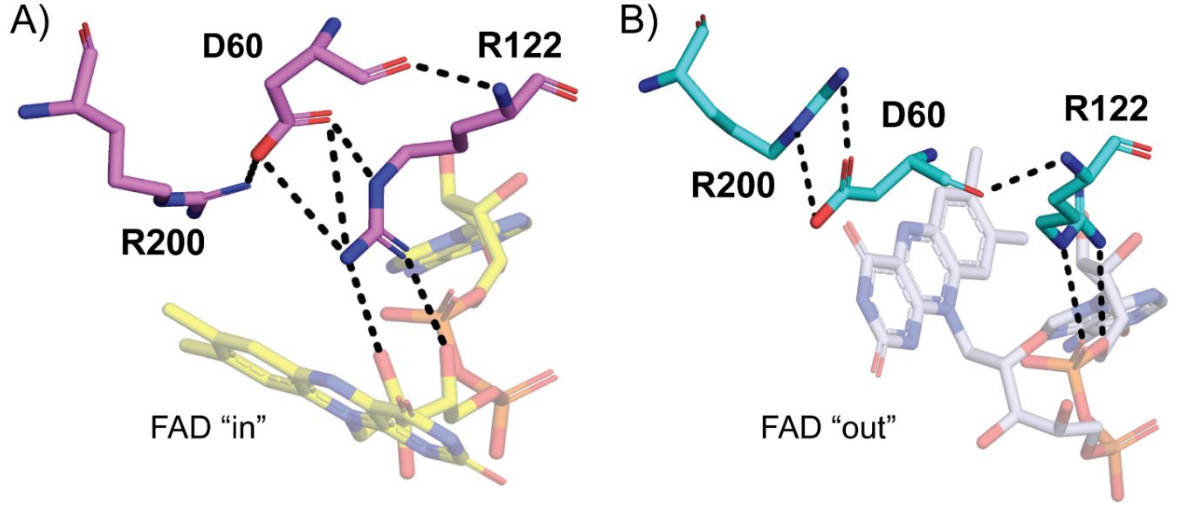

Figure S7. Flavin and active site motion in CtdE. A) Proposed interactions of D60 with R122, R200 (pink), and the flavin “in” (yellow). B) Predicted interactions of D60, R122, and R200 (blue) with the flavin “out” (gray). All interaction distances are 3.8 Å or less. CtdE with the flavin “in” PDB ID: 7KPT and with the flavin “out” PDB ID: 7KPQ.

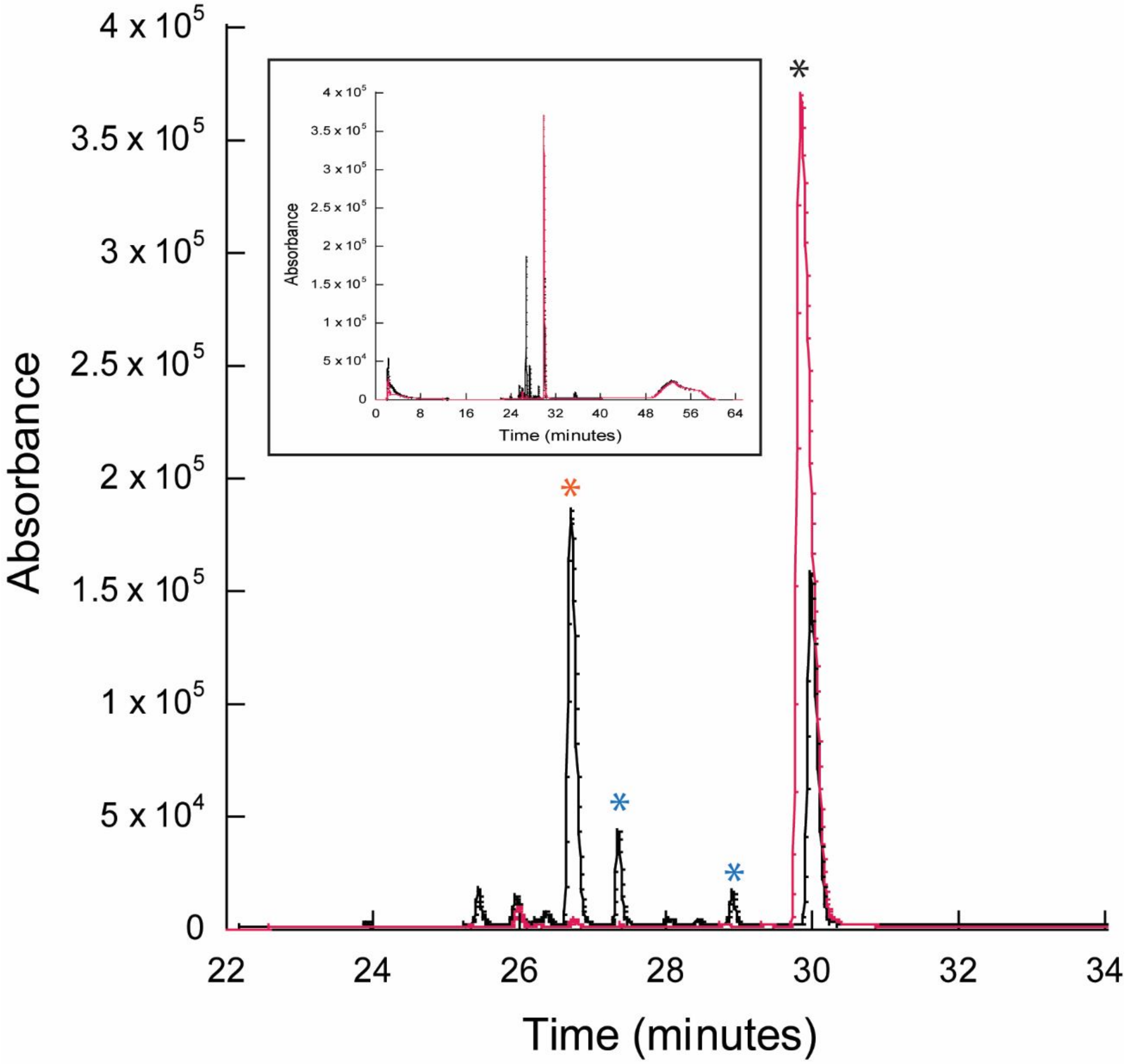

Figure S8. HPLC chromatogram of the product formation assay for D63A. The OxaD trace is in black, and the mutant is in pink. Both the data for OxaD and D63A were collected under the same conditions for direct comparison (1 μM enzyme, 500 μM roquefortine C, and 2.5 mM NADPH). Data was collected at 305 nm. The figure inset displays the entire chromatograms. Peaks indicated with a black asterisk correspond to the substrate, peaks corresponding to NADPH are indicated with an orange asterisk, and peaks corresponding to products are indicated with a blue asterisk.

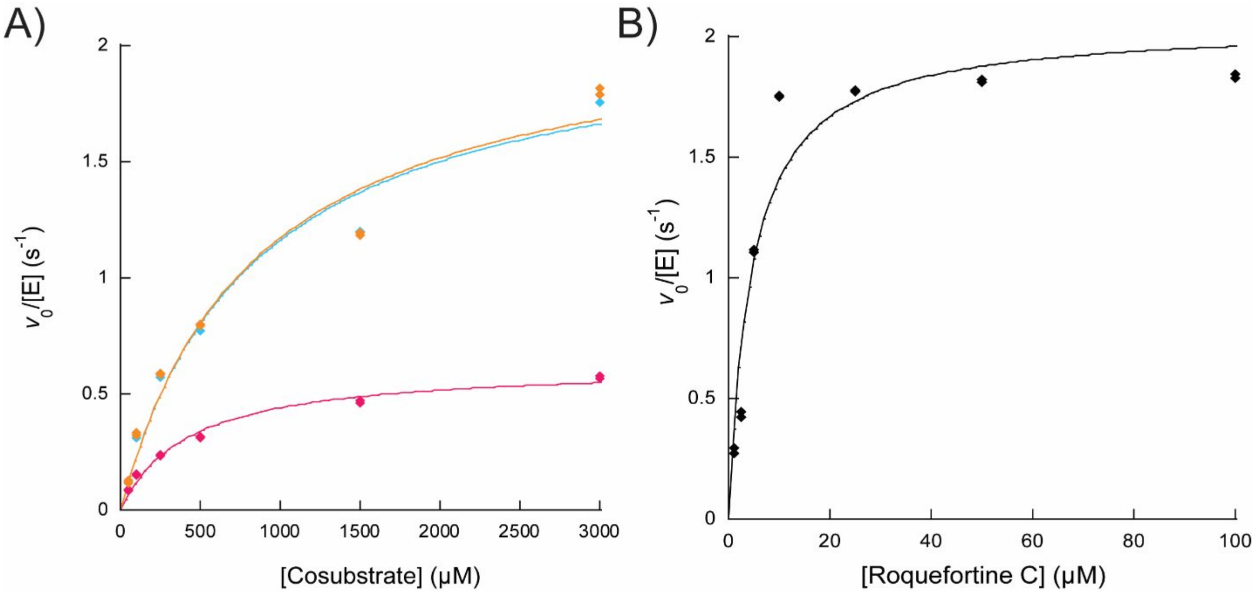

Figure S9. Steady-state kinetic analysis of D63A. A) Activity of as a function of NADPH (blue), synthesized NADPH (orange), and (*R*)-[4-^{2}H]-NADPH (pink). All cosubstrates were tested between 250-3000 μM and roquefortine C was fixed at 100 μM. B) D63A activity with roquefortine C monitoring oxygen consumption. Roquefortine C was varied between 2.5-100 μM and NADPH was fixed at 2500 μM. The data in both panels were fit to Eq. (1). In all experiments, 1 μM enzyme was used.

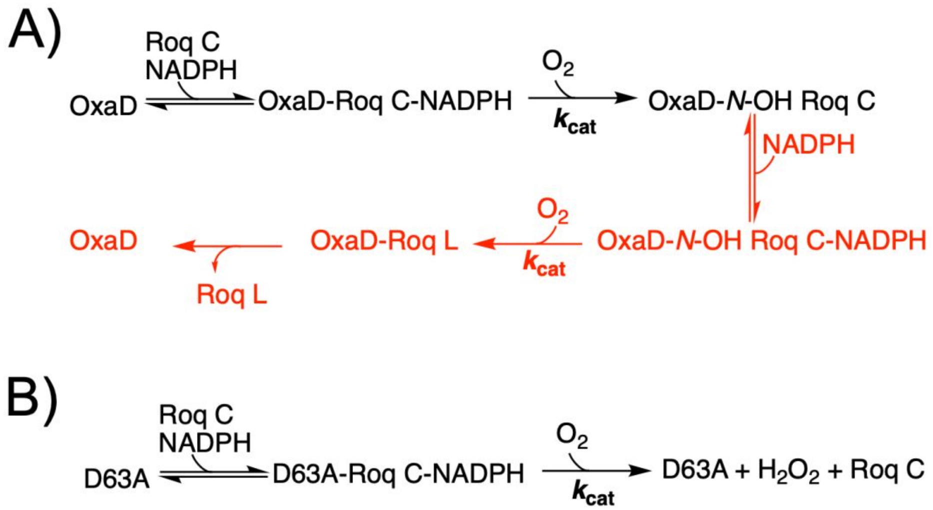

Supplemental Scheme 1. Schematic representations of the predicted rate-contributing steps of OxaD and D63A. A) OxaD catalyzes two successive oxidation reactions of roquefortine C (Roq C) to produce the roquefortine L (Roq L). In each oxidation flavin dehydration is likely the rate-contributing step and therefore the k_{cat} values reported are a combination of this step from each reaction. B) D63A only has NADPH oxidase activity, where reaction uncoupling is the rate-contributing and is the major factor contributing to the k_{cat} value.

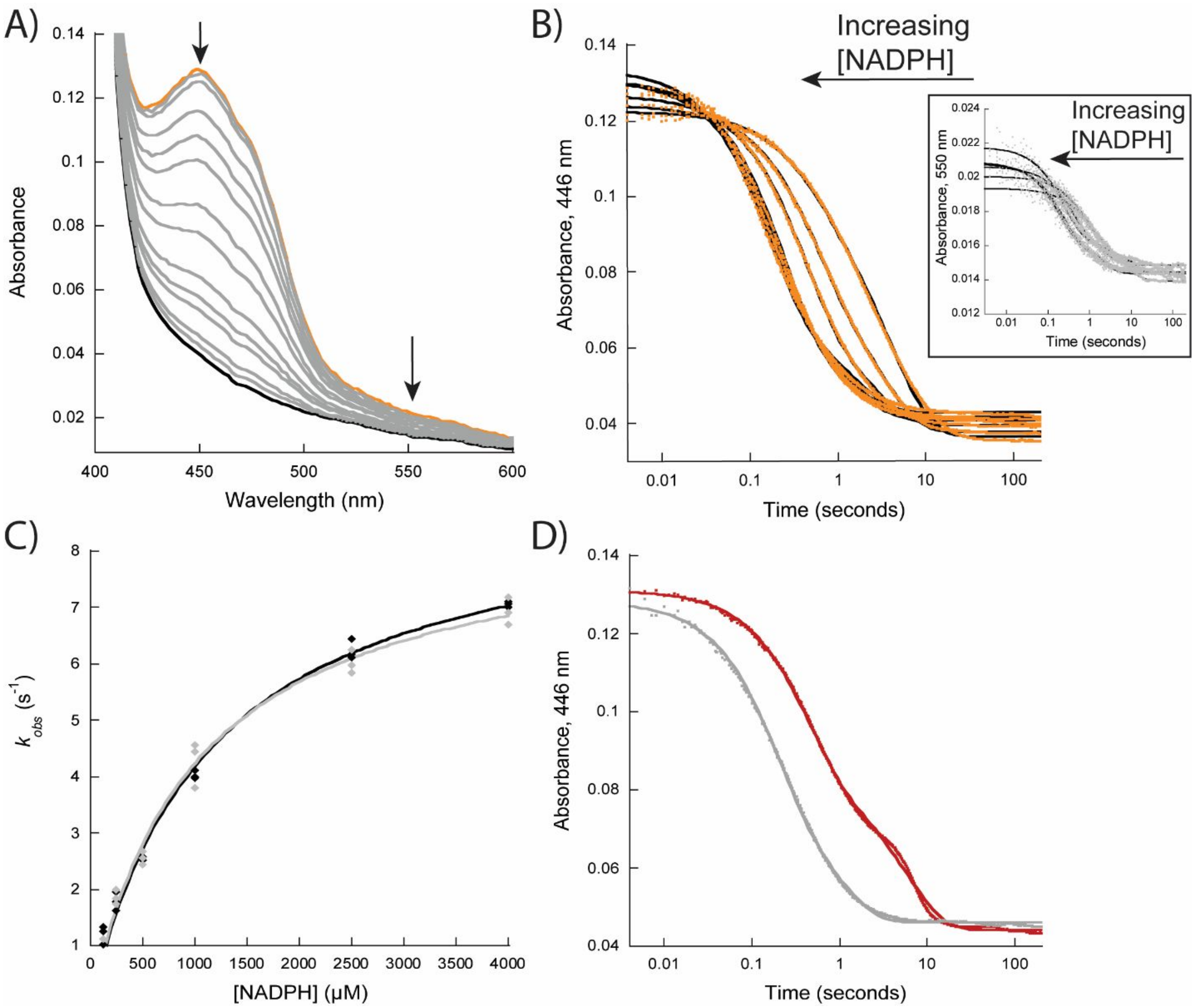

Figure S10. Reduction of D63A. A) Reduction spectra at 4 mM NADPH over 200 seconds. The fully oxidized flavin is shown in orange and the reduced flavin is shown in black. The direction of the changes in absorbance are indicated by the arrows. B) Representative traces of the flavin reduction at 446 nm and absorbance decreases at 550 nm (gray, inset) were fit to Eq. (2). The data fits of both sets of traces are in the black. C) Observed rates resulting from absorbance changes at 446 nm (black) and at 550 nm (gray) were fit to Eq. (3). D) Traces from the kinetic isotope effect experiments. The NADPH reduction trace is shown in gray and the (*R*)-[4-^{2}H]-NADPH is shown in red fit to Eq. (2). Both were at a concentration of 4 mM. For all experiments, the roquefortine C and D63A concentrations were 10 μM.

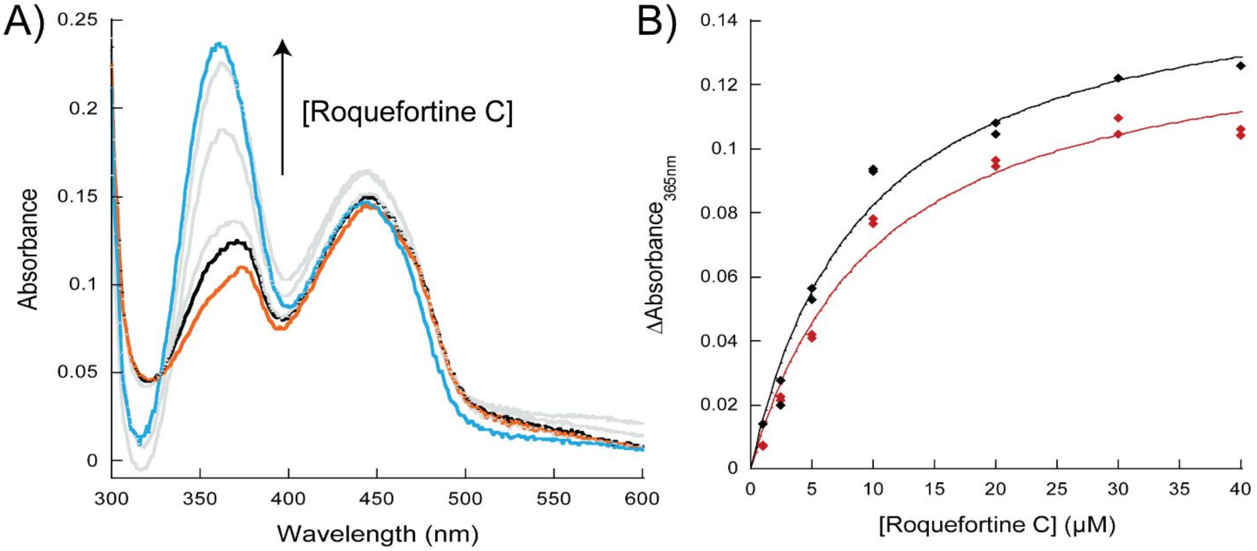

Figure S11. Binding affinity of roquefortine C to OxaD and D63A. A) Spectra of OxaD and various roquefortine C concentrations (1-40 μM). The roquefortine C concentration increases as indicated by the arrow. The spectrum without substrate is in orange, the lowest concentration of roquefortine C tested is in black (1 μM) and the highest is in blue (40 μM). The spectra of D63A from the same experiment look very similar to the one shown in panel A. B) Fitting of the change in absorbance at the unique peak resulting from the enzyme-substrate complex for OxaD (black) and D63A (red) to Eq. (5). The concentration of enzyme used was 15 μM.

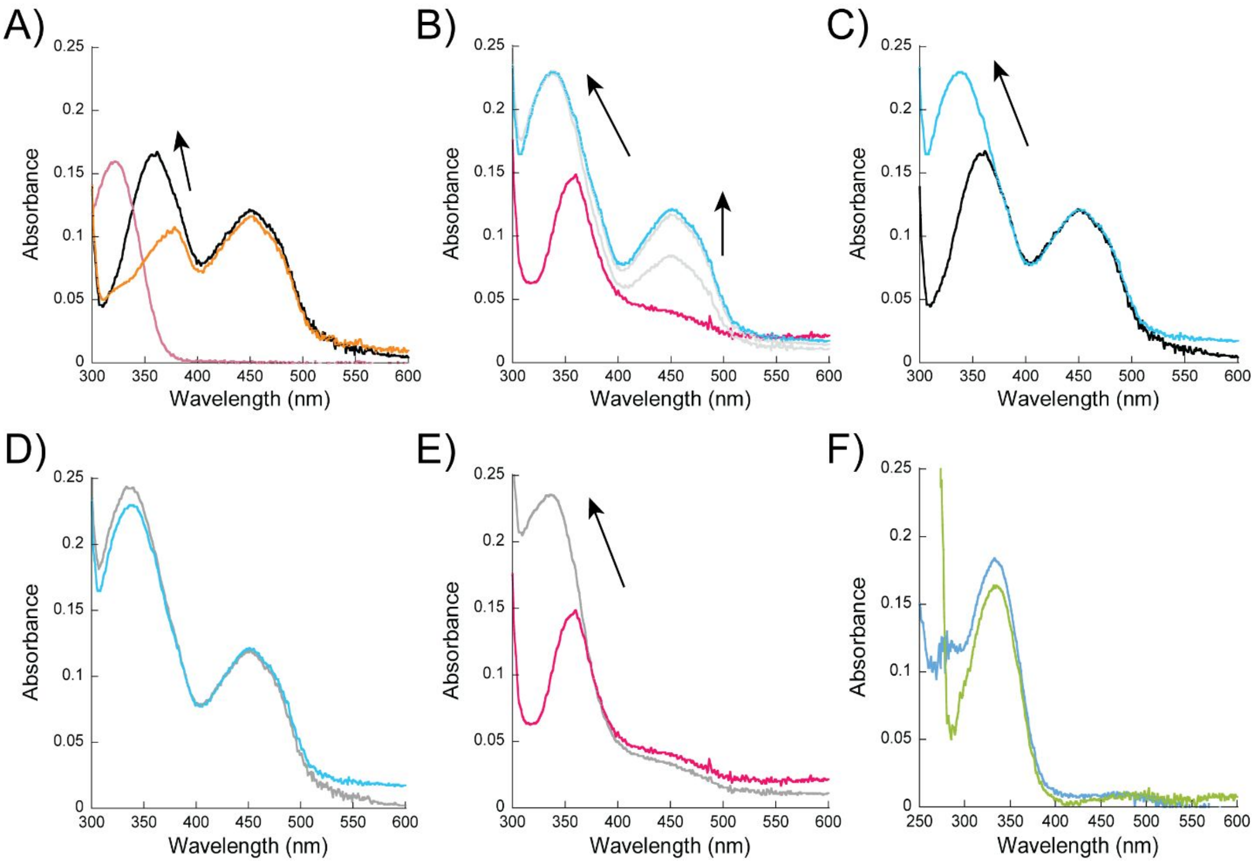

Figure S12. Spectral analysis of D63A. A) Comparison of the spectra of 10 µM roquefortine C (pink) with 10 µM of free enzyme (orange) and 10 µM enzyme in complex with 10 µM roquefortine C (black). B) Spectrum of D63A in the reduced with 20 µM NADPH (pink) and then 2 minutes after exposure to air (blue). Gray spectra represent 30 seconds and 1 minute after air exposure. C) 10 µM enzyme in complex with 10 µM roquefortine C without catalysis occurring (black) and with catalysis occurring (blue). D) Comparison of the enzyme-substrate complex without catalysis after 20 minutes of incubation (gray) to the spectra after catalysis was completed (blue). E) Comparison of the reduced enzyme spectra immediately after reduction (pink) and after 1 hour (gray). F) Subtraction of the free enzyme spectra from the enzyme-substrate complex after oxidation (blue) and after 20 minutes of incubation without catalysis (green). All incubation was conducted at room temperature.

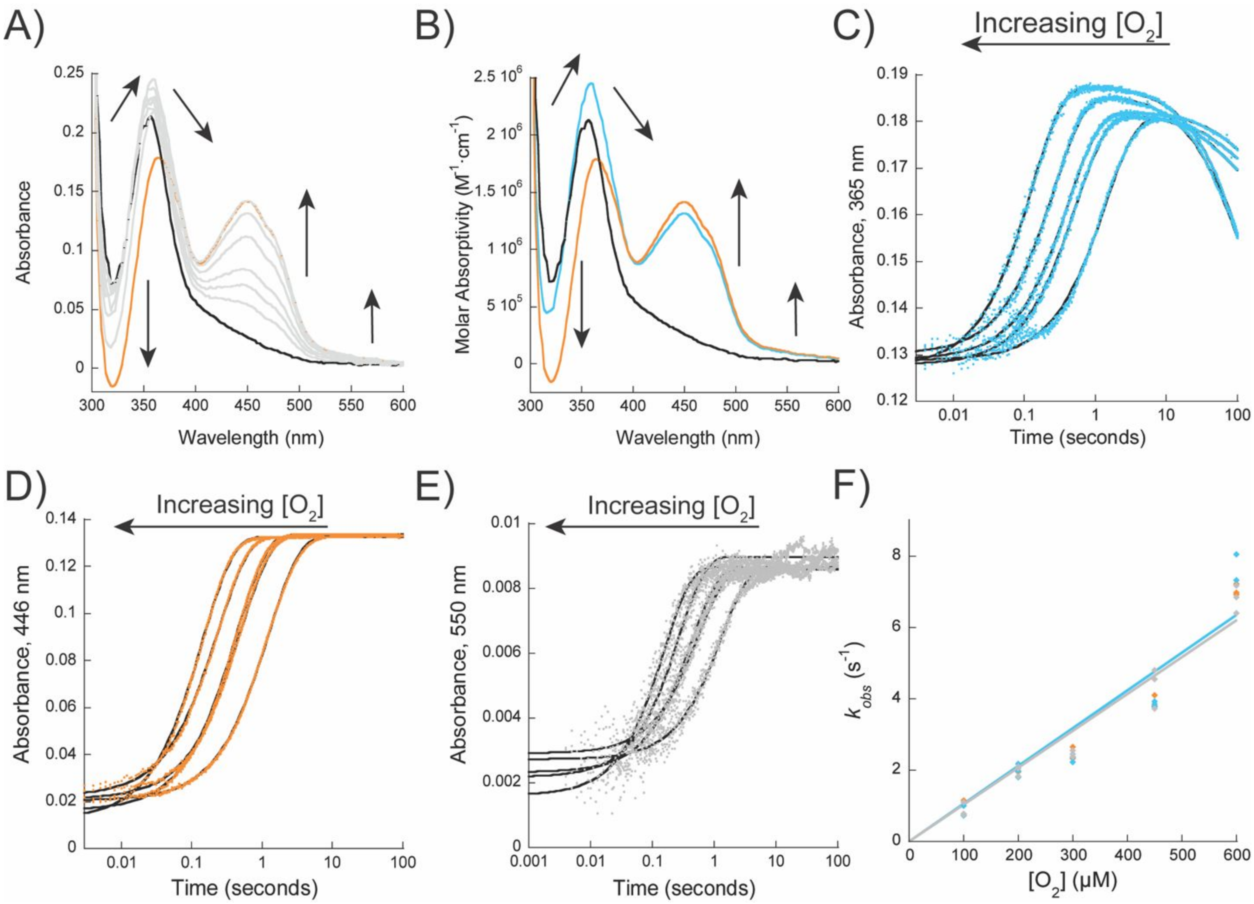

Figure S13. Oxidative-half reaction results for D63A. A) Changes in the spectra of the reduced D63A-roquefortine C upon reaction with 600 μM O_2 over 100 seconds. The reduced enzyme is indicated in black, the oxidized enzyme-substrate complex is shown in blue, and the final oxidation spectra is in in orange. B) The deconvoluted oxidation spectra of the reduced enzyme (black), an intermediate that forms upon reaction of the reduced enzyme with oxygen (blue) and the oxidized flavin (orange). C) Rapid-reaction traces at 365 nm (blue) fit to Eq. (4). These traces represent the formation of an enzyme-substrate complex as oxidation occurs. D) Rapid-reaction traces at 446 nm (orange) fit to Eq. (5). These traces represent the formation of fully oxidized flavin. E) Representative traces at 550 nm (gray) were fit to Eq. (5). In panels C-E, the data fitting is shown in black. F) Determination of bimolecular rate constant for the formation of the enzyme-intermediate complex (blue), and rates of complete flavin oxidation (orange) and rate of increase at 550 nm (gray) as a function oxygen concentration.

Table S1. Rapid-reaction kinetic parameters for the oxidative-half reaction.

Enzyme	k_{oxy} [O_2] ($M^{-1} \cdot s^{-1}$)	k_{OH} [O_2] ($M^{-1} \cdot s^{-1}$)
OxaD	1.8 x 10^4	N.D.
D63A	N.D.	1.1 x 10^4

Conditions: 25 mM HEPES/Na^+ buffer, pH 7.6, 50 mM NaCl, 1% DMSO. All experiments contained 10 μM roquefortine C and enzyme. Error values were obtained from the data fitting analysis.
